# Sensitivity to change of generic preference-based instruments (EQ-5D-3L, EQ-5D-5L, and HUI3) in the context of treatment for people with prescription-type opioid use disorder in Canada

**DOI:** 10.1007/s11136-023-03381-6

**Published:** 2023-04-07

**Authors:** David G. T. Whitehurst, Cassandra Mah, Emanuel Krebs, Benjamin Enns, M. Eugenia Socias, Didier Jutras-Aswad, Bernard Le Foll, Bohdan Nosyk, For the OPTIMA Research Group within the Canadian Research Initiative in Substance Misuse

**Affiliations:** 1 Faculty of Health Sciences, Simon Fraser University, Burnaby, BC Canada; 2 Centre for Health Evaluation and Outcome Sciences, Vancouver, BC Canada; 3 Department of Medicine, Faculty of Medicine, University of British Columbia, Vancouver, BC Canada; 4 British Columbia Centre on Substance Use, Vancouver, BC Canada; 5 Research Centre, Centre Hospitalier de l’Université de Montréal, Montreal, QC Canada; 6 Department of Psychiatry and Addiction, Faculty of Medicine, Université de Montréal, Montreal, QC Canada; 7 Department of Pharmacology and Toxicology, Faculty of Medicine, University of Toronto, Toronto, ON Canada; 8 Department of Family and Community Medicine, Faculty of Medicine, University of Toronto, Toronto, ON Canada; 9 Department of Psychiatry, University of Toronto, Toronto, ON Canada; 10 Dalla Lana School of Public Health, University of Toronto, Toronto, ON Canada; 11 Translational Addiction Research Laboratory, Campbell Family Mental Health Research Institute, Center for Addiction and Mental Health (CAMH), Toronto, ON Canada; 12 Acute Care Program, CAMH, Toronto, ON Canada

**Keywords:** Sensitivity to change, Responsiveness, EQ-5D, Health Utilities Index, Opioid use disorder, Quality-adjusted life years

## Abstract

**Purpose:**

Using data from a randomized controlled trial for treatment of prescription-type opioid use disorder in Canada, this study examines sensitivity to change in three preference-based instruments [EQ-5D-3L, EQ-5D-5L, and the Health Utilities Index Mark 3 (HUI3)] and explores an oft-overlooked consideration when working with contemporaneous responses for similar questions—data quality.

**Methods:**

Analyses focused on the relative abilities of three instruments to capture change in health status. Distributional methods were used to categorize individuals as ‘improved’ or ‘not improved’ for eight anchors (seven clinical, one generic). Sensitivity to change was assessed using area under the ROC (receiver operating characteristics) curve (AUC) analysis and comparisons of mean change scores for three time periods. A ‘strict’ data quality criteria, defined a priori, was applied. Analyses were replicated using ‘soft’ and ‘no’ criteria.

**Results:**

Data from 160 individuals were used in the analysis; 30% had at least one data quality violation at baseline. Despite mean index scores being significantly lower for the HUI3 compared with EQ-5D instruments at each time point, the magnitudes of change scores were similar. No instrument demonstrated superior sensitivity to change. While six of the 10 highest AUC estimates were for the HUI3, ‘moderate’ classifications of discriminative ability were identified in 12 (of 22) analyses for each EQ-5D instrument, compared with eight for the HUI3.

**Conclusion:**

Negligible differences were observed between the EQ-5D-3L, EQ-5D-5L, and HUI3 regarding the ability to measure change. The prevalence of data quality violations—which differed by ethnicity—requires further investigation.

**Supplementary Information:**

The online version contains supplementary material available at 10.1007/s11136-023-03381-6.

## Introduction

Increases in non-medical use of prescription opioids in North America through the early 2000s paralleled the rise in adverse consequences including opioid use disorder, illicit drug use, overdose deaths, and associated societal costs [1, 2]. More recently, these trends have been exacerbated by the availability (through illicit/unregulated sources) of highly potent synthetic opioids such as fentanyl and fentanyl analogue [3]. Within the context of substance use disorders, there is a growing focus on quality of life as an outcome of interest because of the potential to capture the complexities of treatment and recovery [4]. Generic, preference-based health-related quality of life (HRQoL) instruments provide an approach to outcome measurement that can describe health status across person-centered dimensions and incorporate societal valuations of health states. These instruments also allow for the estimation of quality-adjusted life years (QALYs)—the unit of health benefit for economic evaluation recommended by multiple health technology assessment agencies [5, 6].

All preference-based HRQoL instruments comprise a fixed set of items and response options (the classification system), ensuring an individual’s set of responses correspond to one of a finite number of health states. Each instrument has its own scoring procedure (the valuation system)—sometimes multiple, country-specific scoring procedures [7, 8]—providing a set of preference weights that represent the relative value that society places on living in each of the instrument-defined states. While these fundamental components of preference-based HRQoL instruments are alike, differences exist regarding the incorporated dimensions and response options and the methods employed to estimate societal valuations.

The EQ-5D-3L, one of the most widely used preference-based instruments [6, 7], has been used to examine HRQoL trajectories among people with opioid use disorder. Multiple studies have found stable trajectories regardless of retention in opioid agonist treatment [9, 10]. Other work has shown that while initial improvements in HRQoL following initiation of opioid agonist therapy were sustained for some participants, a subpopulation was characterized by an initial improvement that diminished over time [11]. Similar findings have been reported with another preference-based HRQoL instrument, the SF-6D [12]. The EQ-5D-3L has also been shown to be somewhat responsive to decreases in illicit drug use among opioid-dependent patients [13].

Improving the sensitivity of the EQ-5D-3L was part of the rationale when the EuroQol Group embarked on a project that culminated in the EQ-5D-5L [14]. While the composite dimensions remained the same (see ‘Methods’), the EQ-5D-5L expanded the number of response levels per dimension from three to five. It was hypothesized that an increased number of response options would result in a more sensitive instrument, compared with EQ-5D-3L, although evidence across clinical and non-clinical settings is mixed [15–22]. Specific to the context of opioid use disorder, there have been no head-to-head comparisons of preference-based HRQoL instruments that include the EQ-5D-5L. The psychometric performance of the EQ-5D-5L (compared with a capability wellbeing instrument) in a sample of heroin users in opiate substitution treatment was explored in a UK study [23]. Regarding sensitivity to change, there was evidence of a ‘weak’ effect, with the assessment of sensitivity hampered by a ceiling effect, with 26.5% of participants reporting full health at the initial measurement point.

Assessment of psychometric criteria in a head-to-head comparison of preference-based instruments provides valuable insight into the relative performance of the instruments [24]. However, the concurrent administration of instruments comprising similar questions increases the cognitive burden for respondents and the potential for response bias, which may compromise data quality. In this study, we examined sensitivity to change in three preference-based instruments [EQ-5D-3L, EQ-5D-5L, and the Health Utilities Index Mark 3 (HUI3)] in the context of treatment for people with prescription-type opioid use disorder in Canada, incorporating data quality criteria to examine the consistency of participant responses across similar questions.

## Methods

### Data Source & Outcomes

This study is a secondary analysis of data from an open-label, pragmatic, noninferiority randomized controlled trial [25]. The trial was designed to determine whether flexible take-home buprenorphine/naloxone was as effective as supervised methadone in reducing opioid use in people with prescription-type opioid use disorder (where opioids could come from prescriptions or illicit sources). Full details of the trial design and procedures and the clinical and cost-effectiveness results are reported elsewhere [25–27]. Briefly, the clinical report found the buprenorphine/naloxone model of care to be a safe and noninferior alternative to methadone to decrease opioid use, while the economic evaluation concluded buprenorphine/naloxone was not a cost-effective treatment option when evaluated over a lifetime time horizon.

A battery of outcome measures was used in the trial, with data collected at baseline and at follow-up visits every two weeks for 24 weeks (not all instruments were administered at all time points) [26]. Assessments were conducted in-person between October 2017 and March 2020, then via telephone because of the COVID-19 pandemic. Details of the three generic, preference-based HRQoL instruments pertinent to this study (EQ-5D-3L, EQ-5D-5L and HUI3) are provided in Table 1. In addition to the five-dimension classification systems of EQ-5D instruments, the EQ-5D-3L and EQ-5D-5L include a visual analogue scale (EQ VAS), with anchors at 0 (“The worst health you can imagine”) and 100 (“The best health you can imagine”). Responses to the EQ VAS are not used in the derivation of EQ-5D-3L and EQ-5D-5L index scores. The preference-based instruments were administered in a fixed order: EQ-5D-5L was immediately followed by HUI3 and, later in the survey (after completing another 60+ questions), participants completed the EQ-5D-3L.

**Table 1:** Details of the preference-based instruments^a^

Instrument	Dimensions	Number of items used to derive an index score	Number of unique health states	Scoring range for the value set^b^
EQ-5D-3L^c^	5: Mobility, Self-care, Usual Activities, Pain/Discomfort, Anxiety/Depression	5 items, each with 3 response levels	243 (3^5^)	− 0.340 to 1.000 [28]
EQ-5D-5L^c^	5: Mobility, Self-care, Usual Activities, Pain/Discomfort, Anxiety/Depression	5 items, each with 5 response levels	3125 (5^5^)	− 0.148 to 0.949 [29]
HUI3	8: Vision, Hearing, Speech, Ambulation, Dexterity, Emotion, Cognition, Pain	8 items, with either 5 or 6 response levels^d^	972,000 (6^5^ × 5^3^)	− 0.359 to 1.000 [30]

HUI3 indicates Health Utilities Index Mark 3

^a^The EQ-5D-3L, EQ-5D-5L and HUI3 were the only quality of life instruments included in the randomized controlled trial. The rationale for selection was (i) to allow for cost-effectiveness analysis using standard approaches and (ii) to address methodological issues regarding health-related preference-based instruments

^b^Based on the scoring algorithm recommended by the authors of the respective Canadian valuation study. Index scores (also known as health state values) are interpreted on a scale from zero (dead) to one (full health). Negative values represent health states worse than dead

^c^In addition to the five-dimension classification system, EQ-5D instruments include a 0–100 visual analogue scale (EQ VAS). Responses to the EQ VAS are not used in the derivation of index scores

^d^The standard 15-item HUI self-administered questionnaire was not used in this study. Instead, eight questions that comprise the HUI3 classification system were used. This approach has been used in large-scale instrument comparison studies [31]

Other standardized measures used in the analyses (see ‘Statistical Analysis’) assess aspects of mental health, stress, and pain; signs and symptoms of opiate withdrawal; and substance cravings. The Beck Anxiety Inventory and Beck Depression Inventory-II are 21-item self-report inventories for measuring the severity of anxiety and depression, respectively [32, 33]. Each item describes one symptom and responses are provided on a four-point scale, resulting in a scoring range from 0 to 63. Higher scores indicate more severe symptoms. The short form version of the Brief Pain Inventory is a 14-item inventory used to assess pain intensity and the impact of pain on functioning, with a recall period of 24 h [34, 35]. The pain intensity subscale score is the mean of four items, each with responses ranging from 0 (“no pain”) to 10 (“pain as bad as you can imagine”). The pain interference subscale score is the mean of seven items, where participants are asked to rate the degree to which pain has interfered with listed functions, ranging from 0 (“does not interfere”) to 10 (“completely interferes”). The first item of the Brief Pain Inventory asks, “*Throughout our lives, most of us have had pain from time to time (such as minor headaches, sprains, and toothaches). Have you had pain other than these everyday kinds of pain today?*” In the trial, participants answering ‘no’ were assigned subscale scores of zero. The Brief Substance Craving Scale assesses the intensity, frequency, and duration of a craving (each on a five-point scale, where higher scores indicate higher-level cravings) and records the number of times a participant had a craving in the past 24 h [36]. Participants answer the four questions with respect to a primary drug (questions can be repeated for a second craved substance). For each drug/substance, scores for the first three questions can be summed to generate a score from 0 to 12 [37]. In the trial, the primary drug was specified as prescription opioids. Only responses for the primary drug were used in the analysis reported here. The Clinical Opiate Withdrawal Scale is an 11-item clinician-administered assessment tool for measuring withdrawal symptoms [38, 39]. The number of response levels and the scores ascribed to the levels of response vary by item. Total scores range from 0 to 48, with higher scores reflecting more severe withdrawal symptoms. The Kessler Psychological Distress Scale comprises 10 questions about emotional states, each with a five-level response scale, scored from one (“none of the time”) to five (“all of the time”) [40, 41]. Sum scores range from 10 to 50, where higher scores indicate higher levels of psychological distress.

### Defining the Study Sample

Analyses focused on the subset of trial participants who provided complete data (i.e., no missing item-level data) for the preference-based instruments at baseline and *at least one of* the 12-week or 24-week follow-up points. These data defined the maximum number of participants included in the analyses described below, examining change over three time periods: (i) baseline to 12 weeks, (ii) 12 weeks to 24 weeks, and (iii) baseline to 24 weeks. The dataset was further restricted following the application of data quality criteria to identify and remove respondents providing inconsistent (or ‘unreliable’ [42]) responses. A detailed description of the conditions used to define data quality is provided in Supplementary Material 1. To summarize, two conditions (one related to EQ VAS scores, one related to dimension-level EQ-5D-3L and EQ-5D-5L responses) were used to define ‘strict’ and ‘soft’ data quality criteria. Under the strict criteria, a single data quality violation (using either condition) was sufficient to exclude a participant from relevant analyses, whereas the soft criteria excluded participants if there was more than one relevant dimension-level data quality violation. The primary analyses reported here focuses on the dataset created after applying the strict data quality criteria. As a sensitivity analysis, to assess the impact of different ways of defining data quality, all analyses were repeated when applying the soft criteria and no criteria.

### Statistical Analysis

To examine the implications of imposing restrictions on the analytic sample, baseline characteristics for three groups were compared: (i) all trial participants, (ii) the study sample when applying no data quality criteria, and (iii) those in the study sample with at least one data quality violation. Statistical tests (Student’s t-test for age, Pearson’s chi-squared test for nominal variables) were conducted to compare participant characteristics between those with and without at least one data quality violation. Prior to looking at sensitivity to change, descriptive analyses were conducted for the preference-based instruments, comprising descriptive statistics (for index scores and change scores; two-sided paired t-tests for comparisons) and inspection of dimension-level response patterns.

An anchor-based approach was used to examine sensitivity to change. For eight external anchors (seven clinical, one generic), defined using the standardized outcome measures described above, participants were categorized as ‘improved’ or ‘not improved’ in each of the three time periods. None of the measures used as anchors has a published minimum clinically important difference (MCID) estimate in the context of opioid use disorder or substance use. In this study, distributional methods were used to classify changes in the anchor instruments. Based on the observations of Norman and colleagues [43, 44], participants were classified as ‘improved’ if their anchor change score improved by more than half a standard deviation of the sample’s score at the initial time point. ‘Sensitivity to change’ is the chosen term throughout this paper, as opposed to responsiveness or a form of construct validity. Some authors have used such terms interchangeably [21], while others have differentiated between them [45]. Similar to the approach by Goranitis and colleagues [23], our choice of term was driven by the absence of MCID estimates for the anchors used to categorize individuals as ‘improved’ or ‘not improved’.

Each instrument’s ability to discriminate between ‘improved’ and ‘not improved’ groups was assessed in two ways: (i) comparison of group mean change scores (Student’s t-tests) and (ii) area under the ROC (receiver operating characteristics) curve (AUC) analysis. An ROC curve plots the sensitivity (true positive rate) against 1–specificity (1–true negative rate) of classifying participants into binary categories according to varying thresholds of a continuous score. Here, the continuous score is the change in preference-based index score and the binary categories are the two groups defined by the respective anchor. In this context, the AUC represents the ability of the continuous variable to discriminate between groups, with values ranging from zero (perfect incorrect discrimination, where the classifier always predicts the incorrect choice) to 1.00 (perfect correct discrimination). An AUC of 0.50 indicates random detection of change, with no discriminative ability. The AUC was calculated for each instrument/anchor pair, in all three time periods; 95% confidence intervals were generated from 2000 nonparametric, stratified bootstrap samples. Comparisons between instruments were drawn using rule of thumb classifications for AUC estimates (low, between 0.50 and 0.70; moderate, between 0.70 and 0.90; or high, above 0.90) [46, 47] and identification of the 10 highest and 10 lowest estimates.

Analyses were completed using R software version 4.0.5. Statistical tests were interpreted using a significance level of 0.05.

## Results

One hundred sixty (58.8%) of 272 trial participants provided complete data for the EQ-5D-3L, EQ-5D-5L, and HUI3 at baseline and at least one of the 12-week or 24-week follow-up points. Baseline characteristics are reported in Table 2. Ethnicity was the only characteristic that was statistically different between participants in the study sample with (*n* = 90) and without (*n* = 70) at least one data quality violation, where there was a smaller proportion of white participants in the group with at least one violation (see Supplementary Material 2). A breakdown of the number of participants satisfying data quality criteria, by time point, and the frequency of increasing numbers of violations is provided in Supplementary Material 1 (SM1 Table E and SM1 Table D, respectively). Adopting the strict data quality criteria, the maximum sample sizes for analyses at each time point were 112 (baseline), 111 (12 weeks), and 100 (24 weeks); and, for the time periods, 80 (baseline to 12 weeks), 69 (12 weeks to 24 weeks), and 77 (baseline to 24 weeks).

**Table 2:** Baseline characteristics for (i) all trial participants, (ii) the study sample when applying no data quality conditions, and (iii) participants in the study sample with at least one data quality violation. Values are numbers (percentages) unless stated otherwise^a^

Characteristic	Trial participants (*n* = 272)	Study sample: all participants (*n* = 160)	Study sample: ≥ 1 DQ violation (*n* = 90)
Age, mean (SD) years	38.9 (10.6)	40.9 (11.1)	41.9 (11.3)
Gender—man^b^	176 (64.7)	111 (69.4)	60 (66.7)
Highest level of schooling completed			
Middle school or less	50 (18.4)	29 (18.1)	17 (18.9)
High school	118 (43.4)	66 (41.3)	43 (47.8)
Technical/trade school	25 (9.2)	17 (10.6)	10 (11.1)
‘Some’ college/university	32 (11.8)	19 (11.9)	7 (7.8)
College/University	46 (16.9)	29 (18.1)	13 (14.4)
Salary (last 30 days), mean (SD) CAD	620.0 (1472.3)	830.8 (1762.4)	667.8 (1305.5)
Housing stability			
Very unstable	85 (31.2)	41 (25.6)	24 (26.7)
A little unstable	40 (14.7)	27 (16.9)	18 (20.0)
Neither unstable nor stable	22 (8.1)	17 (10.6)	11 (12.2)
A little stable	42 (15.4)	20 (12.5)	7 (7.8)
Very stable	75 (27.6)	51 (31.9)	27 (30.0)
Ethnicity			
White	183 (67.3)	113 (70.6)	56 (62.2)
First Nations or Métis	59 (21.7)	32 (20.0)	23 (25.6)
Asian, Black, Hispanic or other	27 (9.9)	13 (8.1)	10 (11.1)
Lifetime heroin use—yes	187 (68.8)	105 (65.6)	64 (71.1)
Prescription drug coverage—yes	217 (79.8)	135 (84.4)	80 (88.9)
Location of study site (province)			
Alberta	79 (29.0)	30 (18.8)	17 (18.9)
British Columbia	69 (25.4)	37 (23.1)	27 (30.0)
Ontario	52 (19.1)	31 (19.4)	16 (17.8)
Quebec	72 (26.5)	62 (38.8)	30 (33.3)

CAD indicates Canadian dollar; DQ, data quality; HUI3, Health Utilities Index Mark 3; SD, standard deviation

^a^Numbers do not always sum to the respective totals because of missing data or ‘Don’t know’ and ‘Choose not to answer’ responses. Data quality violations were explored at baseline and the 12-week and 24-week follow-up points only

^b^One participant identified as a transgender woman and was included as a woman

Dimension-level response patterns for the EQ-5D-3L, EQ-5D-5L, and HUI3, by time point, are reported in Supplementary Material 3 (SM3 Table A). Consistent observations were that the dimensions with the highest proportions of no impairment (i.e., a level 1 response) were Mobility and Self-care for EQ-5D instruments, and Hearing, Speech, and Dexterity for the HUI3. At all three time points, the highest levels of impairment were reported most frequently for the respective pain and mental health dimensions (Pain/Discomfort and Anxiety/Depression for EQ-5D instruments, Pain and Emotion for the HUI3). The baseline proportions of responses in the highest level of impairment for the pain and mental health dimensions (level 3 for the EQ-5D-3L, level 5 for the EQ-5D-5L and HUI3) were markedly different: 17.9% (EQ-5D-3L), 3.6% (EQ-5D-5L), and 1.8% (HUI3) for the pain items, and 18.8% (EQ-5D-3L), 9.8% (EQ-5D-5L), and 3.6% (HUI3) for the mental health items.

**Fig. 1:**
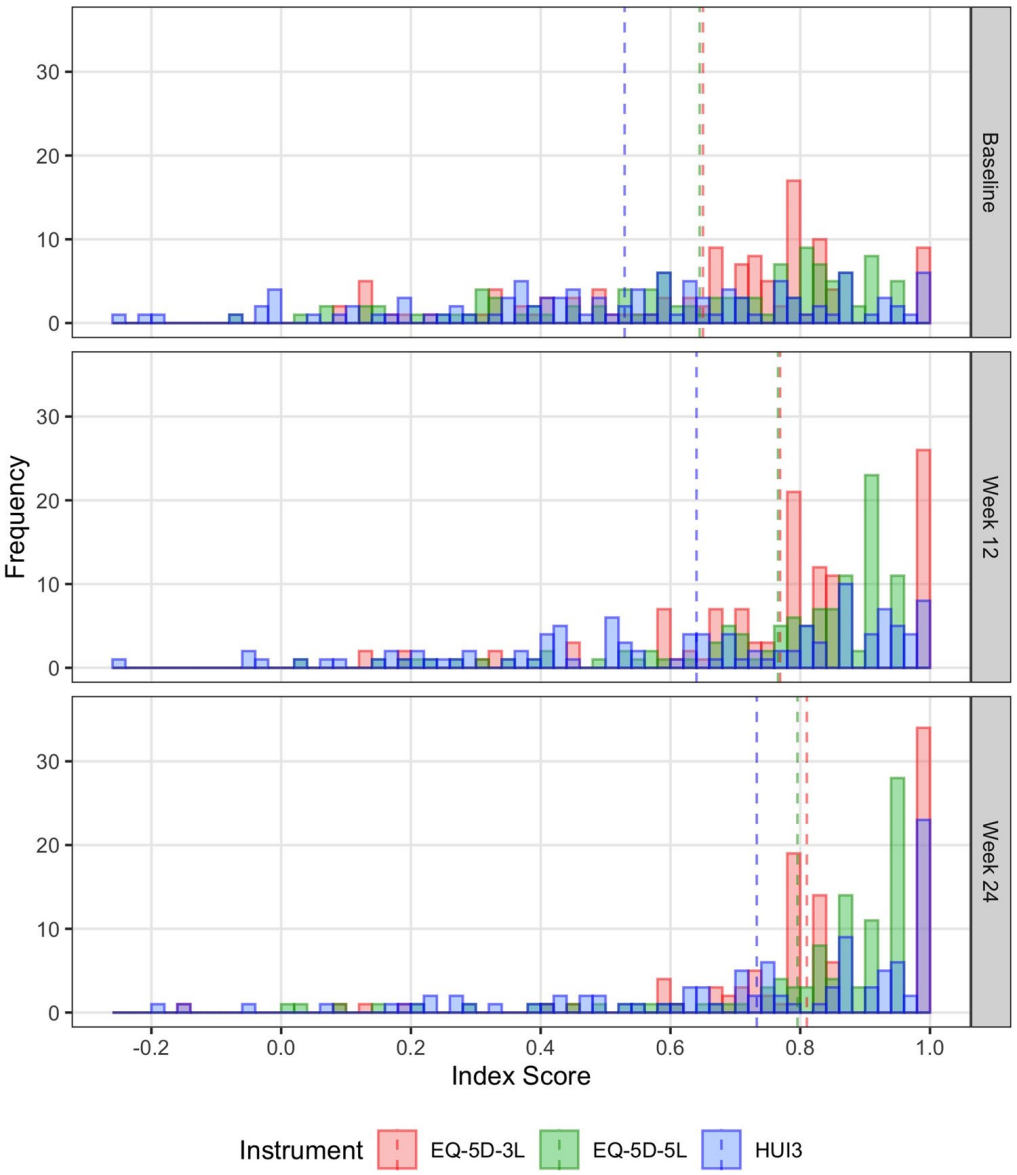
Frequency distributions for EQ-5D-3L, EQ-5D-5L, and HUI3 index scores, by time point, for participants satisfying the strict data quality criteria (baseline, *n* = 112; 12-week follow-up, *n* = 111; 24-week follow-up, *n* = 100). Dashed lines indicate mean values

Figure 1 shows the frequency distributions of index scores, by time point. Descriptive statistics for index scores and change scores are reported in Table 3. At each time point, the HUI3 had the lowest individual value, the lowest mean value (statistically significantly lower than those of EQ-5D instruments) and the widest interquartile range. The magnitudes of change scores across the three time periods were similar. For example, between baseline and 12 weeks, mean change scores were 0.117 (EQ-5D-3L), 0.111 (EQ-5D-5L), and 0.124 (HUI3). There were no statistically significant differences between EQ-5D-3L, EQ-5D-5L, and HUI3 change scores.

**Table 3:** Descriptive statistics for the EQ-5D-3L, EQ-5D-5L, and HUI3 index scores, by time point and time period, for participants satisfying the strict data quality criteria

Instrument	*n*	Mean	SD	Median	IQR	Full health (%)	Negative score (%)	Max.	Min.
Time point/period									
EQ-5D-3L									
Baseline	112	0.650^a^	0.229	0.718	0.269	8.0	0.0	1.000	0.089
Week 12 follow-up	111	0.769^a^	0.197	0.781	0.157	23.4	0.0	1.000	0.122
Week 24 follow-up	100	0.810^a^	0.207	0.826	0.246	34.0	1.0	1.000	− 0.154
Change score (12W-B)	80	0.117	0.185	0.072	0.253	–	–	0.659	− 0.337
Change score (24W-12W)	69	0.056	0.155	0.000	0.118	–	–	0.807	− 0.317
Change score (24W-B)	77	0.140	0.247	0.109	0.291	–	–	0.714	− 0.600
EQ-5D-5L									
Baseline	112	0.645^a^	0.246	0.716	0.302	4.5	0.9	0.949	− 0.066
Week 12 follow-up	111	0.766^a^	0.199	0.829	0.192	9.9	0.0	0.949	0.038
Week 24 follow-up	100	0.796^a^	0.213	0.867	0.184	28.0	0.0	0.949	0.001
Change score (12W-B)	80	0.111	0.179	0.076	0.216	–	–	0.552	− 0.306
Change score (24W-12W)	69	0.032	0.143	0.007	0.090	–	–	0.689	− 0.288
Change score (24W-B)	77	0.121	0.241	0.076	0.243	–	–	0.777	− 0.667
HUI3									
Baseline	112	0.529^b,c^	0.308	0.567	0.416	5.4	8.9	1.000	− 0.257
Week 12 follow-up	111	0.640^b,c^	0.298	0.695	0.457	7.2	3.6	1.000	− 0.243
Week 24 follow-up	100	0.733^b,c^	0.288	0.842	0.328	23.0	3.0	1.000	− 0.187
Change score (12W-B)	80	0.124	0.236	0.129	0.274	–	–	0.829	− 0.443
Change score (24W-12W)	69	0.064	0.200	0.027	0.211	–	–	0.707	− 0.353
Change score (24W-B)	77	0.151	0.276	0.125	0.363	–	–	0.858	− 0.505

12W indicates 12-week follow-up; 24W, 24-week follow-up; B, baseline; HUI3, Health Utilities Index Mark 3; IQR, interquartile range; max., maximum; min., minimum; SD, standard deviation

^a^Statistically different to the corresponding HUI3 index/change score using a significance level of 0.05 (two-sided paired t-test)

^b^Statistically different to the corresponding EQ-5D-3L index/change score using a significance level of 0.05 (two-sided paired t-test)

^c^Statistically different to the corresponding EQ-5D-5L index/change score using a significance level of 0.05 (two-sided paired t-test)

**Fig. 2:**
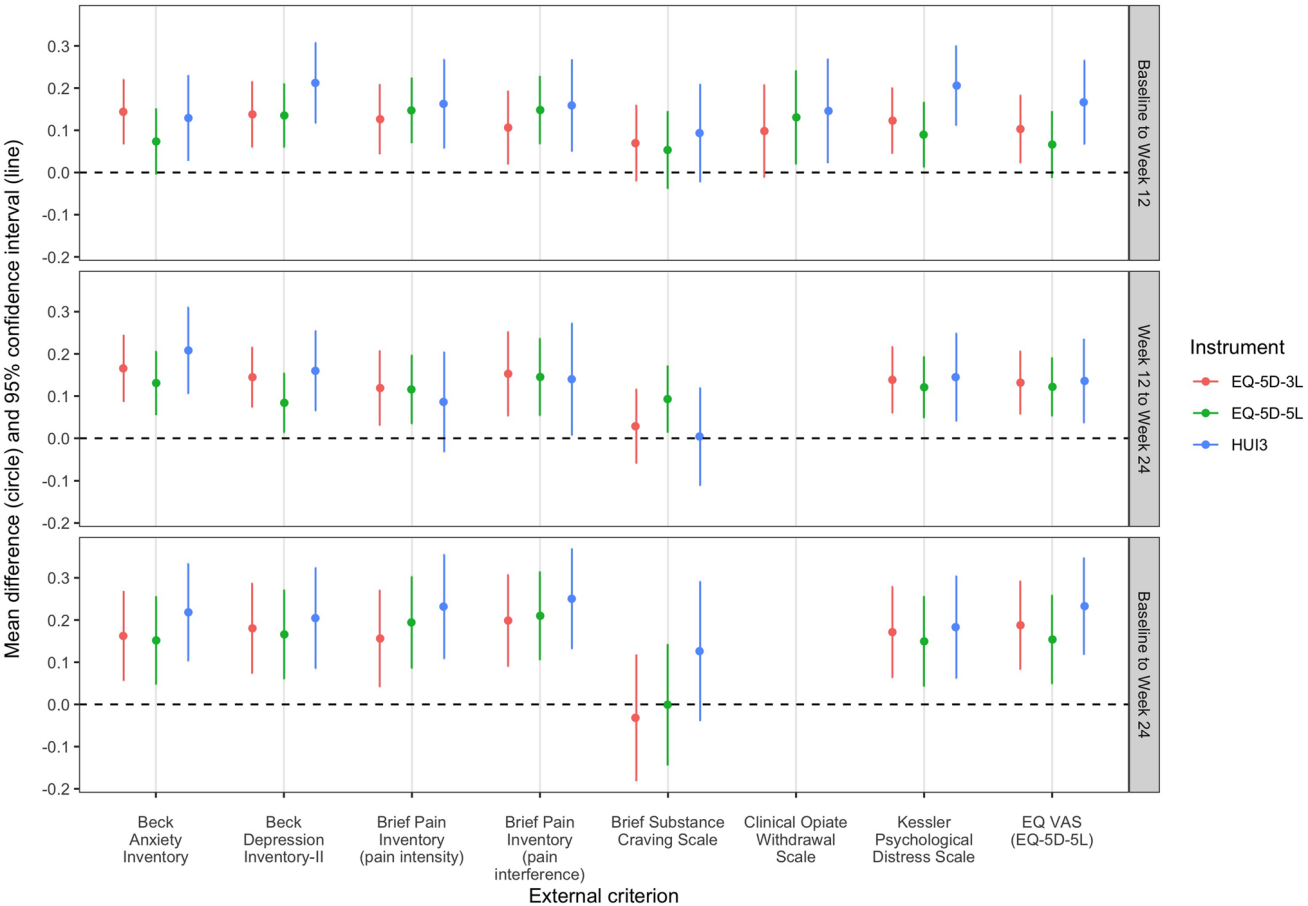
Differences in EQ-5D-3L, EQ-5D-5L, and HUI3 change scores between ‘improved’ and ‘not improved’ groups, by external criterion, for participants satisfying the strict data quality criteria. Results are presented for three time periods. Change scores are calculated as the participant’s index score at the latter time point minus the index score at the former time point. The *difference* in change scores is calculated as the mean value in the ‘improved’ group minus the mean value in the ‘not improved’ group. The number of participants in each group, for each criterion, is reported in Supplementary Material 4

Figure 2 illustrates the results of the mean change score comparisons for the eight anchors (corresponding statistics are provided in Supplementary Material 4). The only anchor with directional inconsistencies, where the change score for the ‘not improved’ group was greater than the change score for the ‘improved’ group, was the Brief Substance Craving Scale. This result was observed for the EQ-5D instruments in the baseline to 24-week time period. The difference in mean change scores for the Brief Substance Craving Scale anchor was non-significant in eight of the nine analyses. For all other anchors—spanning 57 analyses—the differences in change scores were positive and statistically significantly different from zero in 53 analyses. Exceptions were observed for all three preference-based instruments: the Clinical Opiate Withdrawal Scale for the EQ-5D-3L (baseline to 12 weeks), the Beck Anxiety Inventory and EQ VAS for the EQ-5D-5L (baseline to 12 weeks), and the pain intensity subscale of the Brief Pain Inventory for the HUI3 (12 weeks to 24 weeks). Based solely on point estimates, the difference in mean change scores was highest for the HUI3 in 18 of 22 (82%) comparisons.

The 10 lowest AUC estimates mirrored the non-significant change score comparisons, including seven for the Brief Substance Craving Scale anchor (see Table 4). Six of the 10 highest AUC estimates were for the HUI3, three for the EQ-5D-5L and one for the EQ-5D-3L. Six different anchors were present in the highest 10 estimates, with five of the estimates for subscales of the Brief Pain Inventory. There were no ‘high’ classifications of AUC estimates; ‘moderate’ classifications were identified in 12 (of 22) analyses for each of the EQ-5D instruments and eight for the HUI3. Using the lower bound of the confidence intervals as the statistic of interest, no estimates would be classified as ‘moderate’.

**Table 4:** Area under the receiver operating characteristics (ROC) curve analysis, by preference-based instrument and time period, for participants satisfying the strict data quality criteria. Sample size variation within a time period is because of missing data for the external criteria

Time period		Area under the ROC curve (95% confidence interval)		
External criterion	*n*	EQ-5D-3L	EQ-5D-5L	HUI3
Baseline to Week 12				
Beck Anxiety Inventory	80	0.698 (0.58, 0.81)	0.631 (0.51, 0.76)	0.660 (0.54, 0.78)
Beck Depression Inventory-II	79	0.700 (0.59, 0.81)	0.718 (0.60, 0.83)	0.770 (0.66, 0.88)^a^
Brief Pain Inventory—pain intensity	80	0.669 (0.53, 0.81)	0.726 (0.61, 0.85)	0.688 (0.56, 0.81)
Brief Pain Inventory—pain interference	80	0.651 (0.52, 0.78)	0.743 (0.63, 0.86)^a^	0.660 (0.52, 0.80)
Brief Substance Craving Scale^c^	75	0.618 (0.46, 0.78)^b^	0.610 (0.44, 0.78)^b^	0.596 (0.44, 0.75)^b^
Clinical Opiate Withdrawal Scale^d^	53	0.616 (0.46, 0.77)^b^	0.707 (0.56, 0.86)	0.700 (0.54, 0.86)
Kessler Psychological Distress Scale	80	0.702 (0.59, 0.82)	0.671 (0.55, 0.79)	0.784 (0.68, 0.89)^a^
EQ VAS (EQ-5D-5L)	80	0.659 (0.54, 0.78)	0.599 (0.47, 0.73)^b^	0.693 (0.58, 0.81)
Week 12 to Week 24				
Beck Anxiety Inventory	69	0.765 (0.63, 0.90)^a^	0.735 (0.59, 0.88)	0.765 (0.64, 0.89)^a^
Beck Depression Inventory-II	69	0.742 (0.60, 0.88)	0.632 (0.48, 0.78)	0.692 (0.55, 0.83)
Brief Pain Inventory—pain intensity	69	0.666 (0.51, 0.82)	0.711 (0.56, 0.86)	0.564 (0.37, 0.76)^b^
Brief Pain Inventory—pain interference	69	0.709 (0.52, 0.90)	0.751 (0.58, 0.92)^a^	0.643 (0.44, 0.85)
Brief Substance Craving Scale^c^	59	0.478 (0.33, 0.63)^b^	0.622 (0.47, 0.77)	0.496 (0.34, 0.66)^b^
Kessler Psychological Distress Scale	69	0.727 (0.59, 0.87)	0.711 (0.57, 0.85)	0.651 (0.50, 0.80)
EQ VAS (EQ-5D-5L)	69	0.726 (0.60, 0.85)	0.725 (0.58, 0.87)	0.680 (0.54, 0.82)
Baseline to Week 24				
Beck Anxiety Inventory	77	0.704 (0.59, 0.82)	0.647 (0.52, 0.77)	0.740 (0.63, 0.85)
Beck Depression Inventory-II	77	0.731 (0.62, 0.85)	0.696 (0.58, 0.82)	0.682 (0.56, 0.80)
Brief Pain Inventory—pain intensity	77	0.729 (0.61, 0.85)	0.783 (0.67, 0.89)^a^	0.790 (0.68, 0.90)^a^
Brief Pain Inventory—pain interference	77	0.660 (0.53, 0.79)	0.740 (0.63, 0.85)	0.759 (0.65, 0.87)^a^
Brief Substance Craving Scale^c^	68	0.482 (0.30, 0.66)^b^	0.516 (0.34, 0.69)^b^	0.634 (0.47, 0.80)
Kessler Psychological Distress Scale	76	0.726 (0.60, 0.85)	0.701 (0.58, 0.82)	0.658 (0.53, 0.78)
EQ VAS (EQ-5D-5L)	77	0.718 (0.60, 0.83)	0.680 (0.55, 0.81)	0.749 (0.64, 0.86)^a^

CI indicates confidence interval; HUI3, Health Utilities Index Mark 3; VAS, visual analogue scale

^a^One of the highest 10 area under the ROC curve estimates (across all 66 analyses)

^b^One of the lowest 10 area under the ROC curve estimates (across all 66 analyses)

^c^Follow-up for the Brief Substance Craving Scale was at week 10 and week 22 (paired with week 12 and week 24 index scores, respectively)

^d^For the Clinical Opiate Withdrawal Scale criterion, changes in index scores from baseline to week 4 were compared with clinical change measured from treatment initiation (any time from baseline to week 2) to week 4

### Sensitivity analyses

All results obtained after applying the soft criteria and no criteria are reported in the Supplementary Material: dimension-level response patterns (SM3); change scores for ‘improved’ and ‘not improved’ groups, by anchor (SM4); descriptive statistics (SM5); and AUC analysis (SM6). There were no directional inconsistencies in the comparisons of group means. Statistically significant differences were observed in 56 (of 66) analyses using the soft data quality criteria and 55 when using no data quality criteria; in both instances, the Brief Substance Craving Scale anchor accounted for five of the non-significant comparisons. Unlike analyses under the strict data quality criteria, there were statistically significant differences between change scores. The mean HUI3 change score from baseline to 24 weeks was significantly higher than the mean EQ-5D-3L and EQ-5D-5L change scores under the soft criteria, and significantly higher than the mean EQ-5D-3L change score when applying no criteria. Regarding AUC estimates, no ‘high’ classifications were observed in the sensitivity analysis. The number of ‘moderate’ classifications was lower in the sensitivity analyses compared with the strict data criteria for the EQ-5D instruments, and higher for the HUI3. Similar to the strict data criteria analysis, no single instrument dominated the highest 10 AUC estimates.

## Discussion

This study assessed sensitivity to change in the EQ-5D-3L, EQ-5D-5L, and HUI3, over three different time periods, in a sample of people with prescription-type opioid use disorder. Whether comparing group mean estimates or using methods that employ the full distribution of responses, no instrument consistently outperformed the others.

Our finding of no superior instrument aligns with the mixed conclusions from previous comparative analyses that focus on changes in health status [15–22]. Even in studies where authors have identified a ‘better’ instrument—such as a 2022 study by Janssen and colleagues [21]—summary statements are cautious. It should not be unexpected that established instruments, which have each undergone extensive validation in numerous clinical and population samples, perform similarly when measuring change. Contemporaneous, point-in-time estimates can vary widely between preference-based instruments (as seen in Fig. 1) [48, 49], yet such differences between instruments may be negligible when change scores are examined. This emphasizes the need to consider multiple measurement attributes when assessing the relative merits of an instrument (face validity, content validity, construct validity, reliability, etc.)—and, almost inevitably, consideration of ‘better performing instruments’ will require people to make context-specific value judgments. For example, in the context of treatment for prescription-type opioid use disorder, the differences in the distribution of baseline responses to the respective pain and mental health items may discourage further use of the EQ-5D-3L.

Non-significant differences between ‘improved’ and ‘not improved’ groups were more frequent for the condition-specific anchors—eight of nine analyses for the Brief Substance Craving Scale, one of three analyses for the Clinical Opiate Withdrawal Scale—which likely reflects less of a conceptual overlap with the preference-based instruments (compared with domain-specific and generic anchors). The observation of weaker sensitivity of the index scores to changes in the two condition-specific anchors may also be due, partly, to the misaligned administration of measures. For example, Brief Substance Craving Scale scores at week 10 and week 22 were paired with week 12 and week 24 index scores, respectively (exploratory analyses of the trial data has shown the stability of Brief Substance Craving Scale scores from week 6 onward [50]).

The inclusion of near-identical items provided an opportunity to apply data quality checks. At each time point, at least 24% of participants violated a data quality condition; when adopting the strict data quality criteria, sample sizes for analyses by time period were reduced by over 40%. *Specific to this study*, data quality violations did not impact the key findings (i.e., findings were similar across the primary and sensitivity analyses). Definitions of data quality violations—or what other authors have referred to as ‘inconsistencies’ [15, 51, 52, 53] or ‘unreliable answers’ [42]—and the method of reporting violations differ across studies, as do the proportion of individuals or observations failing to satisfy quality/consistency criteria. For example, in a study with cognitively impaired patients living with dementia, Michalowsky and colleagues found at least one dimension-level inconsistency in 64 (49%) of 131 assessments [53]. In the current study, data quality violations were, typically, one-off occurrences for participants (see SM1 Table D), which aligns with similar findings in the contexts of multimorbidity [15] and atopic dermatitis [52].

The observation that data quality violations were more common in racialized participants warrants further investigation. While this statistically significant finding may be spurious—a consequence of multiple testing [54] —it raises questions about acceptance and accessibility of generic standardized instruments. Systematic differences between participants regarding comprehension, cultural relevance and/or communication with research staff are all potential reasons for the observation. Issues of acceptance and accessibility for EQ-5D instruments have been explored in groups such as the Deaf population and people living with aphasia [55, 56]. More broadly, a research program in Australia is developing a preference-based wellbeing measure for Aboriginal and Torres Strait Islander adults, acknowledging that understandings of health and wellbeing are culturally rooted [57]. A common theme across such methodological research is the desire to have instruments that are accessible to a wider community of users, thus improving representation of societal preferences in health care decision making. Further research to investigate the causes and consequences (and definition) of data quality violations, in different clinical and demographic contexts, is complementary to these endeavors.

Strengths of the study include the use of responses to three widely used preference-based HRQoL instruments (including the first use of the HUI3 in the context of opioid use disorder), all with preference weights derived from individuals from the same country (Canada); examination of instruments’ ability to capture change using different methodological approaches (ROC curves and group mean comparisons) across three time periods; and comprehensive consideration of data quality. As with all studies, there are limitations, and caution is advised when generalizing findings beyond the study population. For example, the data-intensive nature of examining change resulted in a significant proportion of participants from the data source (the trial) being excluded from the analysis. The primary limitation was the absence of relevant published MID estimates for the anchors (or questions about self-perceived changes in general health in the study surveys). Instead, distribution-based methods were used, which ensure there are differences in the degree of change between groups but make no judgment about whether these differences are clinically meaningful. In extolling the universality of the ‘half a standard deviation’ distribution-based approach, Norman and colleagues state that it “*could be viewed as a default value* [for reflecting minimal change that matters to patients] *unless other evidence comes to light*” [43]. Another limitation was the administration of instruments in a fixed order, although steps were taken to mitigate question-order bias concerns (i.e., 60 + questions between the EQ-5D instruments). The similarity in participant characteristics between the trial participants (*n* = 272) and the study sample (*n* = 160) (see Table 2) suggests the repetition of questions did not lead to any systematic dropout due to some form of response bias. Finally, as is typical when using data from pragmatic randomized controlled trials, the directionality of changes in health precluded separate examination of sensitivity to positive and negative change.

## Conclusion

Negligible differences were observed between the EQ-5D-3L, EQ-5D-5L, and HUI3 regarding the ability to measure change. While the importance of comparative assessments of ‘similar’ questions/instruments is not in dispute, findings from this study highlight the need for further investigation into the definition, causes, and consequences of data quality violations.

## Supplementary Information

Below is the link to the electronic supplementary material.

Supplementary file1 (DOCX 165 kb)
