# Supplementary material for: Sensitivity to change of generic preference-based instruments (EQ-5D-3L, EQ-5D-5L, and HUI3) in the context of treatment for people with prescription-type opioid use disorder in Canada
Source: Qual Life Res. 2023 Apr 7;32(8):2209–21. doi: 10.1007/s11136-023-03381-6 (PMC10080515; doi:10.1007/s11136-023-03381-6)
Supplement: Supplementary file 1 — Supplementary file1 (DOCX 165 kb) [file 11136_2023_3381_MOESM1_ESM.docx]

**SUPPLEMENTARY MATERIAL**

**Supplementary Material 1 (pages 2-6)**

Details of the data quality conditions and the ‘strict’ and ‘soft’ criteria.

**Supplementary Material 2 (page 7)**

A comparison of the ethnicity breakdowns for the full study sample, participants with at least one data quality violation and participants with no data quality violations

**Supplementary Material 3 (pages 8-16)**

Dimension-level response patterns for the EQ-5D-3L, EQ-5D-5L and HUI3, by time point and data quality criteria.

**Supplementary Material 4 (pages 17-28)**

EQ-5D-3L, EQ-5D-5L and HUI3 change scores for ‘improved’ and ‘not improved’ groups, by external criterion and data quality criteria. Results are presented for three time periods.

**Supplementary Material 5 (pages 29-32)**

Descriptive statistics for the EQ-5D-3L, EQ-5D-5L and HUI3, by time point and time period, for (i) participants satisfying the soft data quality criteria and (ii) the study sample when applying no data quality conditions.

**Supplementary Material 6 (pages 33-36)**

Area under the receiver operating characteristic (ROC) curve analysis, by preference-based instrument and time period, for (i) participants satisfying the soft data quality criteria and (ii) the study sample when applying no data quality conditions.

**Supplementary Material 1**

Consideration of data quality, at each time point, comprised the following conditions:

- **VAS condition —** scores on the EQ VAS (EQ-5D-3L) and EQ VAS (EQ-5D-5L) must be within 50 points of each other
- **Dimension-level condition** — assuming the three levels of the EQ-5D-3L approximate the five levels of the EQ-5D-5L insomuch as,
  - EQ-5D-3L level 1 ≈ EQ-5D-5L level 1,
  - EQ-5D-3L level 2 ≈ EQ-5D-5L level 3, and
  - EQ-5D-3L level 3 ≈ EQ-5D-5L level 5,

an EQ-5D-3L dimension-level response must be within one level of the approximated EQ-5D-5L level. An exception to this rule was made for the Mobility dimension. Because of the wording for the level 3 response for EQ-5D-3L Mobility (“*I am confined to bed*”), a level 2 response for EQ-5D-3L Mobility (“*some problems in walking about*”) was regarded as consistent – for the purposes of a data quality check – with levels 2, 3, 4 and 5 of the EQ-5D-5L (as opposed to levels 2, 3 and 4 only).

The dimension-level condition is illustrated in **SM1 Figure A**, using Mobility and Self-care as examples. Green cells meet the dimension-level condition, red cells do not. Using Mobility and Self-care as examples highlights the different rule used for the Mobility dimension. Crosstabulations of EQ-5D-3L and EQ-5D-5L dimension-level responses are provided in **SM1 Table A** (baseline), **SM1 Table B** (12-week follow-up) and **SM1 Table C** (24-week follow-up).

| **SM1 Figure A.** Illustration of the dimension-level data quality condition using the Mobility and Self-care items of the EQ-5D-3L and EQ-5D-5L. Green cells meet the dimension-level condition (as described in SM1), red cells do not. | | | | | | | | | |
| --- | --- | --- | --- | --- | --- | --- | --- | --- | --- |
|  |  | EQ-5D-3L Mobility | | |  |  | EQ-5D-3L Self-care | | |
|  |  | L1 | L2 | L3 |  |  | L1 | L2 | L3 |
| EQ-5D-5L Mobility | L1 |  |  |  | EQ-5D-5L Self-care | L1 |  |  |  |
|  | L2 |  |  |  |  | L2 |  |  |  |
|  | L3 |  |  |  |  | L3 |  |  |  |
|  | L4 |  |  |  |  | L4 |  |  |  |
|  | L5 |  |  |  |  | L5 |  |  |  |
| L indicates level. | | | | | | | | | |

| **SM1 Table A.** Crosstabulation of EQ-5D-3L and EQ-5D-5L dimension-level responses at baseline (n=160). Values are numbers (percentages).^a^ | | | | | |
| --- | --- | --- | --- | --- | --- |
| EQ-5D-5L  EQ-5D-3L | No problems | Slight problems | Moderate problems | Severe problems | Unable to/ Extreme problems |
| Mobility |  |  |  |  |  |
| No problems | 80 (50.0) | 12 (7.5) | **2 (1.2)** | **0 (0.0)** | **0 (0.0)** |
| Some problems | **3 (1.9)** | 22 (13.8) | 27 (16.9) | 10 (6.2) | 1 (0.6) |
| Unable to | **0 (0.0)** | **0 (0.0)** | **2 (1.2)** | 0 (0.0) | 1 (0.6) |
| Self-care |  |  |  |  |  |
| No problems | 116 (72.5) | 6 (3.8) | **4 (2.5)** | **0 (0.0)** | **0 (0.0)** |
| Some problems | **2 (1.2)** | 15 (9.4) | 10 (6.2) | 6 (3.8) | **0 (0.0)** |
| Unable to | **0 (0.0)** | **0 (0.0)** | **0 (0.0)** | 0 (0.0) | 1 (0.6) |
| Usual activities |  |  |  |  |  |
| No problems | 49 (30.6) | 18 (11.2) | **4 (2.5)** | **0 (0.0)** | **0 (0.0)** |
| Some problems | **6 (3.8)** | 32 (20.0) | 27 (16.9) | 12 (7.5) | **2 (1.2)** |
| Unable to | **0 (0.0)** | **1 (0.6)** | **0 (0.0)** | 7 (4.4) | 2 (1.2) |
| Pain/Discomfort |  |  |  |  |  |
| No problems | 29 (18.1) | 7 (4.4) | **2 (1.2)** | **1 (0.6)** | **0 (0.0)** |
| Some problems | **3 (1.9)** | 22 (13.8) | 51 (31.9) | 11 (6.9) | **3 (1.9)** |
| Extreme problems | **0 (0.0)** | **0 (0.0)** | **1 (0.6)** | 20 (12.5) | 10 (6.2) |
| Anxiety/Depression |  |  |  |  |  |
| No problems | 15 (9.4) | 12 (7.5) | **6 (3.8)** | **0 (0.0)** | **1 (0.6)** |
| Some problems | **8 (5.0)** | 25 (15.6) | 46 (28.7) | 10 (6.2) | **2 (1.2)** |
| Extreme problems | **0 (0.0)** | **0 (0.0)** | **6 (3.8)** | 13 (8.1) | 16 (10.0) |
| ^a^ The wording used to describe the levels of each dimension (e.g., ‘no problems’, ‘unable to’, etc.) are general descriptors used in EQ-5D User Guides, *not* the dimension-specific wording for each level. Cells with text in **bold red** indicate the dimension-level condition violations, as described in SM1. | | | | | |

| **SM1 Table B.** Crosstabulation of EQ-5D-3L and EQ-5D-5L dimension-level responses at 12-week follow-up (n=146). Values are numbers (percentages).^a^ | | | | | |
| --- | --- | --- | --- | --- | --- |
| EQ-5D-5L  EQ-5D-3L | No problems | Slight problems | Moderate problems | Severe problems | Unable to/ Extreme problems |
| Mobility |  |  |  |  |  |
| No problems | 89 (61.0) | 10 (6.8) | **3 (2.1)** | **1 (0.7)** | **0 (0.0)** |
| Some problems | **2 (1.4)** | 18 (12.3) | 12 (8.2) | 10 (6.8) | 0 (0.0) |
| Unable to | **0 (0.0)** | **1 (0.7)** | **0 (0.0)** | 0 (0.0) | 0 (0.0) |
| Self-care |  |  |  |  |  |
| No problems | 102 (69.9) | 14 (9.6) | **1 (0.7)** | **3 (2.1)** | **0 (0.0)** |
| Some problems | **3 (2.1)** | 13 (8.9) | 9 (6.2) | 1 (0.7) | **0 (0.0)** |
| Unable to | **0 (0.0)** | **0 (0.0)** | **0 (0.0)** | 0 (0.0) | 0 (0.0) |
| Usual activities |  |  |  |  |  |
| No problems | 68 (46.6) | 19 (13.0) | **4 (2.7)** | **1 (0.7)** | **0 (0.0)** |
| Some problems | **8 (5.5)** | 23 (15.8) | 17 (11.6) | 3 (2.1) | **1 (0.7)** |
| Unable to | **0 (0.0)** | **1 (0.7)** | **0 (0.0)** | 0 (0.0) | 1 (0.7) |
| Pain/Discomfort |  |  |  |  |  |
| No problems | 40 (27.4) | 11 (7.5) | **2 (1.4)** | **1 (0.7)** | **0 (0.0)** |
| Some problems | **2 (1.4)** | 32 (21.9) | 34 (23.3) | 5 (3.4) | **1 (0.7)** |
| Extreme problems | **0 (0.0)** | **2 (1.4)** | **2 (1.4)** | 7 (4.8) | 7 (4.8) |
| Anxiety/Depression |  |  |  |  |  |
| No problems | 30 (20.5) | 17 (11.6) | **1 (0.7)** | **0 (0.0)** | **0 (0.0)** |
| Some problems | **3 (2.1)** | 32 (21.9) | 39 (26.7) | 6 (4.1) | **0 (0.0)** |
| Extreme problems | **0 (0.0)** | **2 (1.4)** | **5 (3.4)** | 4 (2.7) | 7 (4.8) |
| ^a^ The wording used to describe the levels of each dimension (e.g., ‘no problems’, ‘unable to’, etc.) are general descriptors used in EQ-5D User Guides, *not* the dimension-specific wording for each level. Cells with text in **bold red** indicate the dimension-level condition violations, as described in SM1. | | | | | |

| **SM1 Table C.** Crosstabulation of EQ-5D-3L and EQ-5D-5L dimension-level responses at 24-week follow-up (n=136). Values are numbers (percentages).^a^ | | | | | |
| --- | --- | --- | --- | --- | --- |
| EQ-5D-5L  EQ-5D-3L | No problems | Slight problems | Moderate problems | Severe problems | Unable to/ Extreme problems |
| Mobility |  |  |  |  |  |
| No problems | 92 (67.6) | 6 (4.4) | **1 (0.7)** | **0 (0.0)** | **0 (0.0)** |
| Some problems | **1 (0.7)** | 11 (8.1) | 15 (11.0) | 8 (5.9) | 1 (0.7) |
| Unable to | **0 (0.0)** | **0 (0.0)** | **0 (0.0)** | 0 (0.0) | 1 (0.7) |
| Self-care |  |  |  |  |  |
| No problems | 108 (79.4) | 4 (2.9) | **1 (0.7)** | **1 (0.7)** | **0 (0.0)** |
| Some problems | **5 (3.7)** | 9 (6.6) | 5 (3.7) | 3 (2.2) | **0 (0.0)** |
| Unable to | **0 (0.0)** | **0 (0.0)** | **0 (0.0)** | 0 (0.0) | 0 (0.0) |
| Usual activities |  |  |  |  |  |
| No problems | 79 (58.1) | 15 (11.0) | **1 (0.7)** | **1 (0.7)** | **0 (0.0)** |
| Some problems | **5 (3.7)** | 14 (10.3) | 14 (10.3) | 5 (3.7) | **0 (0.0)** |
| Unable to | **0 (0.0)** | **0 (0.0)** | **1 (0.7)** | 1 (0.7) | 0 (0.0) |
| Pain/Discomfort |  |  |  |  |  |
| No problems | 49 (36.0) | 11 (8.1) | **0 (0.0)** | **1 (0.7)** | **0 (0.0)** |
| Some problems | **5 (3.7)** | 23 (16.9) | 23 (16.9) | 6 (4.4) | **2 (1.5)** |
| Extreme problems | **1 (0.7)** | **0 (0.0)** | **3 (2.2)** | 8 (5.9) | 4 (2.9) |
| Anxiety/Depression |  |  |  |  |  |
| No problems | 47 (34.6) | 6 (4.4) | **1 (0.7)** | **0 (0.0)** | **0 (0.0)** |
| Some problems | **5 (3.7)** | 33 (24.3) | 23 (16.9) | 6 (4.4) | **0 (0.0)** |
| Extreme problems | **0 (0.0)** | **0 (0.0)** | **4 (2.9)** | 4 (2.9) | 7 (5.1) |
| ^a^ The wording used to describe the levels of each dimension (e.g., ‘no problems’, ‘unable to’, etc.) are general descriptors used in EQ-5D User Guides, *not* the dimension-specific wording for each level. Cells with text in **bold red** indicate the dimension-level condition violations, as described in SM1. | | | | | |

The frequency of increasing numbers of violations of the dimension-level condition, by time point, is reported in **SM1 Table D**. At each time point, at least 70% of respondents satisfied the dimension-level condition, and the highest number of violations at a given time point was three. Violations of the VAS condition were less common than violations of the dimension-level condition. There were two VAS violations at baseline (one of these participants also had a dimension-level violation at baseline), none at 12-week follow-up, and four at 24-week follow-up (none of these participants had a dimension-level violation at 24-week follow-up).

| **SM1 Table D.** Frequency of violations of the dimension-level condition, by time point. Values are numbers (percentages). | | | |
| --- | --- | --- | --- |
| Number of violations | Baseline  (n=160) | Week 12 follow-up (n=146) | Week 24 follow-up (n=136) |
| 0 | 112 (70.0) | 111 (76.0) | 104 (76.5) |
| 1 | 39 (24.4) | 22 (15.1) | 25 (18.4) |
| 2 | 7 (4.4) | 11 (7.5) | 7 (5.1) |
| 3 | 2 (1.3) | 2 (1.4) | 0 (0.0) |
| 4+ | 0 (0.0) | 0 (0.0) | 0 (0.0) |

*‘Strict’ and ‘soft’ data quality criteria*

The primary analyses reported in the paper were performed on a data set created after applying **‘strict’ data quality criteria**. Under this criteria, a single data quality violation (whether the VAS condition or the dimension-level condition) was sufficient to exclude a participant from relevant analyses. For example, a respondent with one data quality violation only (such as reporting EQ-5D-5L level 1 and EQ-5D-3L level 2 for the Anxiety/Depression dimension), at 12-week follow-up, would be excluded from all analyses focusing on the ‘baseline to 12 weeks’ and ‘12 weeks to 24 weeks’ time periods. [Note that such an individual would remain eligible for inclusion in analyses focusing on the ‘baseline to 24 weeks’ time period.] The **‘soft’ data quality criteria**, which was explored in the sensitivity analyses, excluded participants if there was *more than one* relevant dimension-level data quality violation. For example, a respondent with one dimension-level data quality violation at each time point (baseline, 12-week follow-up, and 24-week follow-up) would not be excluded from any analyses under the soft data quality criteria.

The number of participants satisfying the dimension-level and VAS data quality conditions for each time point and time period is reported in **SM1 Table E**.

| **SM1 Table E.** Number (percentage) of participants satisfying data quality conditions for each time point and time period. | | |
| --- | --- | --- |
| Time point/period (n, when applying no data  quality conditions) | Strict criteria | Soft criteria |
| Baseline (n=160) | 112 (70.0) | 149 (93.1) |
| Week 12 follow-up (n=146) | 111 (76.0) | 133 (91.1) |
| Week 24 follow-up (n=136) | 100 (73.5) | 125 (91.9) |
| Baseline to Week 12 follow-up (n=146) | 80 (54.8) | 125 (85.6) |
| Week 12 follow-up to Week 24 follow-up (n=122) | 69 (56.6) | 103 (84.4) |
| Baseline to Week 24 follow-up (n=136) | 77 (56.6) | 117 (86.0) |

**Supplementary Material 2**

The table below (SM2 Table A) replicates the ethnicity breakdown for the full study sample and participants with at least one data quality violation. For ease of interpretation, the breakdown for participants with no data quality violations has been added.

| **SM2 Table A.** Ethnicity breakdown for (i) all participants in the study sample, (ii) participants with at least one data quality violation and (iii) participants with no data quality violations. Values are numbers (percentages). | | | |
| --- | --- | --- | --- |
| Ethnicity | Study sample:  all participants (n=160) | Study sample:  ≥1 DQ violation  (n=90) | Study sample:  no DQ violations  (n=70) |
| White | 113 (70.6) | 56 (62.2) | 57 (81.4) |
| First Nations or Métis | 32 (20.0) | 23 (25.6) | 9 (12.9) |
| Asian, Black, Hispanic or other | 13 (8.1) | 10 (11.1) | 3 (4.3) |
| Chose not to answer^a^ | 2 (1.3) | 1 (1.1) | 1 (1.4) |
| DQ indicates data quality | | | |
| ^a^ This category was not included in the Pearson’s chi-squared test | | | |

Ninety (56.3%) of the 160 participants in the study sample had at least one data quality violation, and the proportion of people with at least one violation was systematically different across the three ethnicity categories reported in SM2 Table 2: white, First Nations or Métis, and ‘Asian, Black, Hispanic or other’ (due to small numbers of responses, several response categories were collapsed to create the third category). Almost half (56/113, 49.6%) of white participants had at least one data quality violation, while the corresponding proportion approached three quarters for non-white participants (23/32, 71.9% for First Nations or Metis; and 10/13, 76.9% for ‘Asian, Black, Hispanic or other’). The statistical significance of the difference in proportions was confirmed with a Pearson’s chi-squared test (*χ^2^* = 7.4915, *df* = 2, *p* = 0.024).

**Supplementary Material 3**

| **SM3 Table A**. Dimension-level response patterns for the EQ-5D-3L, EQ-5D-5L and HUI3, by time point, for participants satisfying the strict data quality criteria. Values are numbers (percentages). | | | | | | |
| --- | --- | --- | --- | --- | --- | --- |
| Instrument: time point | Level 1 | Level 2 | Level 3 | Level 4 | Level 5 | Level 6 |
| Dimension |  |  |  |  |  |  |
| EQ-5D-3L: baseline (n=112) | | | | | | |
| Mobility | 69 (61.6) | 42 (37.5) | 1 (0.9) | - | - | - |
| Self-care | 90 (80.4) | 21 (18.8) | 1 (0.9) | - | - | - |
| Usual Activities | 50 (44.6) | 55 (49.1) | 7 (6.3) | - | - | - |
| Pain/Discomfort | 26 (23.2) | 66 (58.9) | 20 (17.9) | - | - | - |
| Anxiety/Depression | 23 (20.5) | 68 (60.7) | 21 (18.8) | - | - | - |
| EQ-5D-5L: baseline (n=112) | | | | | | |
| Mobility | 63 (56.3) | 19 (17.0) | 21 (18.8) | 8 (7.1) | 1 (0.9) | - |
| Self-care | 87 (77.7) | 12 (10.7) | 6 (5.4) | 6 (5.4) | 1 (0.9) | - |
| Usual Activities | 35 (31.3) | 42 (37.5) | 19 (17.0) | 15 (13.4) | 1 (0.9) | - |
| Pain/Discomfort | 21 (18.8) | 24 (21.4) | 38 (33.9) | 25 (22.3) | 4 (3.6) | - |
| Anxiety/Depression | 13 (11.6) | 32 (28.6) | 37 (33.0) | 19 (17.0) | 11 (9.8) | - |
| HUI3: baseline (n=112) | | | | | | |
| Vision | 57 (50.9) | 36 (32.1) | 7 (6.3) | 7 (6.3) | 4 (3.6) | 1 (0.9) |
| Hearing | 99 (88.4) | 4 (3.6) | 4 (3.6) | 4 (3.6) | 0 (0.0) | 1 (0.9) |
| Speech | 97 (86.6) | 9 (8.0) | 4 (3.6) | 2 (1.8) | 0 (0.0) | - |
| Ambulation | 80 (71.4) | 24 (21.4) | 4 (3.6) | 1 (0.9) | 2 (1.8) | 1 (0.9) |
| Dexterity | 91 (81.3) | 13 (11.6) | 4 (3.6) | 3 (2.7) | 1 (0.9) | 0 (0.0) |
| Emotion | 18 (16.1) | 32 (28.6) | 40 (35.7) | 18 (16.1) | 4 (3.6) | - |
| Cognition | 49 (43.8) | 19 (17.0) | 25 (22.3) | 11 (9.8) | 7 (6.3) | 1 (0.9) |
| Pain | 20 (17.9) | 28 (25.0) | 33 (29.5) | 29 (25.9) | 2 (1.8) | - |
| EQ-5D-3L: Week 12 follow-up (n=111) | | | | | | |
| Mobility | 81 (73.0) | 30 (27.0) | 0 (0.0) | - | - | - |
| Self-care | 94 (84.7) | 17 (15.3) | 0 (0.0) | - | - | - |
| Usual Activities | 76 (68.5) | 34 (30.6) | 1 (0.9) | - | - | - |
| Pain/Discomfort | 42 (37.8) | 61 (55.0) | 8 (7.2) | - | - | - |
| Anxiety/Depression | 41 (36.9) | 63 (56.8) | 7 (6.3) | - | - | - |
| EQ-5D-5L: Week 12 follow-up (n=111) | | | | | | |
| Mobility | 72 (64.9) | 25 (22.5) | 8 (7.2) | 6 (5.4) | 0 (0.0) | - |
| Self-care | 84 (75.7) | 19 (17.1) | 7 (6.3) | 1 (0.9) | 0 (0.0) | - |
| Usual Activities | 63 (56.8) | 31 (27.9) | 13 (11.7) | 3 (2.7) | 1 (0.9) | - |
| Pain/Discomfort | 34 (30.6) | 36 (32.4) | 28 (25.2) | 11 (9.9) | 2 (1.8) | - |
| Anxiety/Depression | 26 (23.4) | 43 (38.7) | 31 (27.9) | 7 (6.3) | 4 (3.6) | - |
| HUI3: Week 12 follow-up (n=111) | | | | | | |
| Vision | 63 (56.8) | 35 (31.5) | 6 (5.4) | 5 (4.5) | 2 (1.8) | 0 (0.0) |
| Hearing | 88 (79.3) | 15 (13.5) | 2 (1.8) | 5 (4.5) | 1 (0.9) | 0 (0.0) |
| Speech | 93 (83.8) | 13 (11.7) | 5 (4.5) | 0 (0.0) | 0 (0.0) | - |
| Ambulation | 88 (79.3) | 18 (16.2) | 4 (3.6) | 1 (0.9) | 0 (0.0) | 0 (0.0) |
| Dexterity | 89 (80.2) | 13 (11.7) | 5 (4.5) | 3 (2.7) | 0 (0.0) | 1 (0.9) |
| Emotion | 30 (27.0) | 32 (28.8) | 40 (36.0) | 7 (6.3) | 2 (1.8) | - |
| Cognition | 54 (48.6) | 19 (17.1) | 24 (21.6) | 11 (9.9) | 3 (2.7) | 0 (0.0) |
| Pain | 36 (32.4) | 30 (27.0) | 27 (24.3) | 15 (13.5) | 3 (2.7) | - |
| EQ-5D-3L: Week 24 follow-up (n=100) | | | | | | |
| Mobility | 81 (81.0) | 18 (18.0) | 1 (1.0) | - | - | - |
| Self-care | 89 (89.0) | 11 (11.0) | 0 (0.0) | - | - | - |
| Usual Activities | 79 (79.0) | 20 (20.0) | 1 (1.0) | - | - | - |
| Pain/Discomfort | 53 (53.0) | 41 (41.0) | 6 (6.0) | - | - | - |
| Anxiety/Depression | 45 (45.0) | 50 (50.0) | 5 (5.0) | - | - | - |
| EQ-5D-5L: Week 24 follow-up (n=100) | | | | | | |
| Mobility | 75 (75.0) | 14 (14.0) | 5 (5.0) | 4 (4.0) | 2 (2.0) | - |
| Self-care | 86 (86.0) | 9 (9.0) | 3 (3.0) | 2 (2.0) | 0 (0.0) | - |
| Usual Activities | 66 (66.0) | 22 (22.0) | 8 (8.0) | 4 (4.0) | 0 (0.0) | - |
| Pain/Discomfort | 44 (44.0) | 28 (28.0) | 16 (16.0) | 9 (9.0) | 3 (3.0) | - |
| Anxiety/Depression | 39 (39.0) | 35 (35.0) | 16 (16.0) | 6 (6.0) | 4 (4.0) | - |
| HUI3: Week 24 follow-up (n=100) | | | | | | |
| Vision | 67 (67.0) | 28 (28.0) | 2 (2.0) | 1 (1.0) | 2 (2.0) | 0 (0.0) |
| Hearing | 91 (91.0) | 7 (7.0) | 2 (2.0) | 0 (0.0) | 0 (0.0) | 0 (0.0) |
| Speech | 93 (93.0) | 7 (7.0) | 0 (0.0) | 0 (0.0) | 0 (0.0) | - |
| Ambulation | 83 (83.0) | 13 (13.0) | 3 (3.0) | 1 (1.0) | 0 (0.0) | 0 (0.0) |
| Dexterity | 85 (85.0) | 10 (10.0) | 3 (3.0) | 2 (2.0) | 0 (0.0) | 0 (0.0) |
| Emotion | 38 (38.0) | 35 (35.0) | 18 (18.0) | 7 (7.0) | 2 (2.0) | - |
| Cognition | 66 (66.0) | 18 (18.0) | 8 (8.0) | 4 (4.0) | 4 (4.0) | 0 (0.0) |
| Pain | 41 (41.0) | 27 (27.0) | 22 (22.0) | 7 (7.0) | 3 (3.0) | - |
| HUI3 indicates Health Utilities Index Mark 3. | | | | | | |

| **SM3 Table B**. Dimension-level response patterns for the EQ-5D-3L, EQ-5D-5L and HUI3, by time point, for participants satisfying the soft data quality criteria. Values are numbers (percentages). | | | | | | |
| --- | --- | --- | --- | --- | --- | --- |
| Instrument: time point | Level 1 | Level 2 | Level 3 | Level 4 | Level 5 | Level 6 |
| Dimension |  |  |  |  |  |  |
| EQ-5D-3L: baseline (n=149) | | | | | | |
| Mobility | 86 (57.7) | 60 (40.3) | 3 (2.0) | - | - | - |
| Self-care | 116 (77.9) | 32 (21.5) | 1 (0.7) | - | - | - |
| Usual Activities | 65 (43.6) | 74 (49.7) | 10 (6.7) | - | - | - |
| Pain/Discomfort | 36 (24.2) | 84 (56.4) | 29 (19.5) | - | - | - |
| Anxiety/Depression | 30 (20.1) | 85 (57.0) | 34 (22.8) | - | - | - |
| EQ-5D-5L: baseline (n=149) | | | | | | |
| Mobility | 77 (51.7) | 32 (21.5) | 28 (18.8) | 10 (6.7) | 2 (1.3) | - |
| Self-care | 109 (73.2) | 20 (13.4) | 13 (8.7) | 6 (4.0) | 1 (0.7) | - |
| Usual Activities | 49 (32.9) | 50 (33.6) | 27 (18.1) | 19 (12.8) | 4 (2.7) | - |
| Pain/Discomfort | 29 (19.5) | 28 (18.8) | 52 (34.9) | 31 (20.8) | 9 (6.0) | - |
| Anxiety/Depression | 21 (14.1) | 37 (24.8) | 52 (34.9) | 22 (14.8) | 17 (11.4) | - |
| HUI3: baseline (n=149) | | | | | | |
| Vision | 76 (51.0) | 49 (32.9) | 7 (4.7) | 10 (6.7) | 6 (4.0) | 1 (0.7) |
| Hearing | 129 (86.6) | 9 (6.0) | 5 (3.4) | 5 (3.4) | 0 (0.0) | 1 (0.7) |
| Speech | 124 (83.2) | 15 (10.1) | 8 (5.4) | 2 (1.3) | 0 (0.0) | - |
| Ambulation | 107 (71.8) | 31 (20.8) | 7 (4.7) | 1 (0.7) | 2 (1.3) | 1 (0.7) |
| Dexterity | 124 (83.2) | 17 (11.4) | 4 (2.7) | 3 (2.0) | 1 (0.7) | 0 (0.0) |
| Emotion | 22 (14.8) | 38 (25.5) | 50 (33.6) | 32 (21.5) | 7 (4.7) | - |
| Cognition | 57 (38.3) | 28 (18.8) | 33 (22.1) | 19 (12.8) | 10 (6.7) | 2 (1.3) |
| Pain | 24 (16.1) | 38 (25.5) | 41 (27.5) | 39 (26.2) | 7 (4.7) | - |
| EQ-5D-3L: Week 12 follow-up (n=133) | | | | | | |
| Mobility | 96 (72.2) | 37 (27.8) | 0 (0.0) | - | - | - |
| Self-care | 110 (82.7) | 23 (17.3) | 0 (0.0) | - | - | - |
| Usual Activities | 86 (64.7) | 45 (33.8) | 2 (1.5) | - | - | - |
| Pain/Discomfort | 51 (38.3) | 67 (50.4) | 15 (11.3) | - | - | - |
| Anxiety/Depression | 45 (33.8) | 74 (55.6) | 14 (10.5) | - | - | - |
| EQ-5D-5L: Week 12 follow-up (n=133) | | | | | | |
| Mobility | 88 (66.2) | 26 (19.5) | 10 (7.5) | 9 (6.8) | 0 (0.0) | - |
| Self-care | 97 (72.9) | 26 (19.5) | 9 (6.8) | 1 (0.8) | 0 (0.0) | - |
| Usual Activities | 71 (53.4) | 38 (28.6) | 19 (14.3) | 3 (2.3) | 2 (1.5) | - |
| Pain/Discomfort | 39 (29.3) | 42 (31.6) | 34 (25.6) | 12 (9.0) | 6 (4.5) | - |
| Anxiety/Depression | 30 (22.6) | 50 (37.6) | 39 (29.3) | 8 (6.0) | 6 (4.5) | - |
| HUI3: Week 12 follow-up (n=133) | | | | | | |
| Vision | 78 (58.6) | 38 (28.6) | 8 (6.0) | 6 (4.5) | 3 (2.3) | 0 (0.0) |
| Hearing | 107 (80.5) | 17 (12.8) | 2 (1.5) | 6 (4.5) | 1 (0.8) | 0 (0.0) |
| Speech | 113 (85.0) | 15 (11.3) | 5 (3.8) | 0 (0.0) | 0 (0.0) | - |
| Ambulation | 105 (78.9) | 21 (15.8) | 5 (3.8) | 2 (1.5) | 0 (0.0) | 0 (0.0) |
| Dexterity | 108 (81.2) | 15 (11.3) | 5 (3.8) | 4 (3.0) | 0 (0.0) | 1 (0.8) |
| Emotion | 35 (26.3) | 38 (28.6) | 45 (33.8) | 11 (8.3) | 4 (.03) | - |
| Cognition | 63 (47.4) | 24 (18.0) | 28 (21.1) | 13 (9.8) | 5 (3.8) | 0 (0.0) |
| Pain | 42 (31.6) | 32 (24.1) | 34 (25.6) | 19 (14.3) | 6 (4.5) | - |
| EQ-5D-3L: Week 24 follow-up (n=125) | | | | | | |
| Mobility | 97 (78.6) | 27 (21.6) | 1 (0.8) | - | - | - |
| Self-care | 107 (85.6) | 18 (14.4) | 0 (0.0) | - | - | - |
| Usual Activities | 91 (72.8) | 33 (26.4) | 1 (0.8) | - | - | - |
| Pain/Discomfort | 60 (48.0) | 54 (43.2) | 11 (8.8) | - | - | - |
| Anxiety/Depression | 51 (40.8) | 63 (50.4) | 11 (8.8) | - | - | - |
| EQ-5D-5L: Week 24 follow-up (n=125) | | | | | | |
| Mobility | 91 (72.8) | 17 (13.6) | 10 (8.0) | 5 (4.0) | 2 (1.6) | - |
| Self-care | 108 (86.4) | 11 (8.8) | 3 (2.4) | 3 (2.4) | 0 (0.0) | - |
| Usual Activities | 82 (65.6) | 26 (20.8) | 11 (8.8) | 6 (4.8) | 0 (0.0) | - |
| Pain/Discomfort | 53 (42.4) | 34 (27.2) | 22 (17.6) | 12 (9.6) | 4 (3.2) | - |
| Anxiety/Depression | 47 (37.6) | 39 (31.2) | 26 (20.8) | 7 (5.6) | 6 (4.8) | - |
| HUI3: Week 24 follow-up (n=125) | | | | | | |
| Vision | 82 (65.6) | 35 (28.0) | 3 (2.4) | 3 (2.4) | 2 (1.6) | 0 (0.0) |
| Hearing | 111 (88.8) | 11 (8.8) | 2 (1.6) | 1 (0.8) | 0 (0.0) | 0 (0.0) |
| Speech | 113 (90.4) | 12 (9.6) | 0 (0.0) | 0 (0.0) | 0 (0.0) | - |
| Ambulation | 100 (80.0) | 20 (16.0) | 3 (2.4) | 2 (1.6) | 0 (0.0) | 0 (0.0) |
| Dexterity | 103 (82.4) | 16 (12.8) | 3 (2.4) | 3 (2.4) | 0 (0.0) | 0 (0.0) |
| Emotion | 43 (34.4) | 41 (32.8) | 25 (20.0) | 12 (9.6) | 4 (3.2) | - |
| Cognition | 82 (65.6) | 20 (16.0) | 12 (9.6) | 5 (4.0) | 6 (4.8) | 0 (0.0) |
| Pain | 47 (37.6) | 32 (25.6) | 30 (24) | 10 (8.0) | 6 (4.8) | - |
| HUI3 indicates Health Utilities Index Mark 3. | | | | | | |

| **SM3 Table C**. Dimension-level response patterns for the EQ-5D-3L, EQ-5D-5L and HUI3, by time point, for the study sample when applying no data quality conditions. Values are numbers (percentages). | | | | | | |
| --- | --- | --- | --- | --- | --- | --- |
| Instrument: time point | Level 1 | Level 2 | Level 3 | Level 4 | Level 5 | Level 6 |
| Dimension |  |  |  |  |  |  |
| EQ-5D-3L: baseline (n=160) | | | | | | |
| Mobility | 94 (58.8) | 63 (39.4) | 3 (1.9) | - | - | - |
| Self-care | 126 (78.8) | 33 (20.6) | 1 (0.6) | - | - | - |
| Usual Activities | 71 (44.4) | 79 (49.4) | 10 (6.3) | - | - | - |
| Pain/Discomfort | 39 (24.4) | 90 (56.3) | 31 (19.4) | - | - | - |
| Anxiety/Depression | 34 (21.3) | 91 (56.9) | 35 (21.9) | - | - | - |
| EQ-5D-5L: baseline (n=160) | | | | | | |
| Mobility | 83 (51.9) | 34 (21.3) | 31 (19.4) | 10 (6.3) | 2 (1.3) | - |
| Self-care | 118 (73.8) | 21 (13.1) | 14 (8.8) | 6 (3.8) | 1 (0.6) | - |
| Usual Activities | 55 (34.4) | 51 (31.9) | 31 (19.4) | 19 (11.9) | 4 (2.5) | - |
| Pain/Discomfort | 32 (20.0) | 29 (18.1) | 54 (33.8) | 32 (20) | 13 (8.1) | - |
| Anxiety/Depression | 23 (14.4) | 37 (23.1) | 58 (36.3) | 23 (14.4) | 19 (11.9) | - |
| HUI3: baseline (n=160) | | | | | | |
| Vision | 83 (51.9) | 50 (31.3) | 10 (6.3) | 10 (6.3) | 6 (3.8) | 1 (0.6) |
| Hearing | 137 (85.6) | 11 (6.9) | 6 (3.8) | 5 (3.1) | 0 (0.0) | 1 (0.6) |
| Speech | 132 (82.5) | 17 (10.6) | 9 (5.6) | 2 (1.3) | 0 (0.0) | - |
| Ambulation | 116 (72.5) | 33 (20.6) | 7 (4.4) | 1 (0.6) | 2 (1.3) | 1 (0.6) |
| Dexterity | 134 (83.8) | 18 (11.3) | 4 (2.5) | 3 (1.9) | 1 (0.6) | 0 (0.0) |
| Emotion | 24 (15.0) | 42 (26.3) | 53 (33.1) | 33 (20.6) | 8 (5.0) | - |
| Cognition | 64 (40.0) | 30 (18.8) | 35 (21.9) | 19 (11.9) | 10 (6.3) | 2 (1.3) |
| Pain | 27 (16.9) | 41 (25.6) | 42 (26.3) | 42 (26.3) | 8 (5.0) | - |
| EQ-5D-3L: Week 12 follow-up (n=146) | | | | | | |
| Mobility | 103 (70.5) | 42 (28.8) | 1 (0.7) | - | - | - |
| Self-care | 120 (82.2) | 26 (17.8) | 0 (0.0) | - | - | - |
| Usual Activities | 92 (63.0) | 52 (35.6) | 2 (1.4) | - | - | - |
| Pain/Discomfort | 54 (37.0) | 74 (50.7) | 18 (12.3) | - | - | - |
| Anxiety/Depression | 48 (32.9) | 80 (54.8) | 18 (12.3) | - | - | - |
| EQ-5D-5L: Week 12 follow-up (n=146) | | | | | | |
| Mobility | 91 (62.3) | 29 (19.9) | 15 (10.3) | 11 (7.5) | 0 (0.0) | - |
| Self-care | 105 (71.9) | 27 (18.5) | 10 (6.8) | 4 (2.7) | 0 (0.0) | - |
| Usual Activities | 76 (52.1) | 43 (29.5) | 21 (14.4) | 4 (2.7) | 2 (1.4) | - |
| Pain/Discomfort | 42 (28.8) | 45 (30.8) | 38 (26.0) | 13 (8.9) | 8 (5.5) | - |
| Anxiety/Depression | 33 (22.6) | 51 (34.9) | 45 (30.8) | 10 (6.8) | 7 (4.8) | - |
| HUI3: Week 12 follow-up (n=146) | | | | | | |
| Vision | 86 (58.9) | 41 (28.1) | 8 (5.5) | 7 (4.8) | 4 (2.7) | 0 (0.0) |
| Hearing | 116 (79.5) | 19 (13.0) | 2 (1.4) | 7 (4.8) | 2 (1.4) | 0 (0.0) |
| Speech | 124 (84.9) | 16 (11.0) | 6 (4.1) | 0 (0.0) | 0 (0.0) | - |
| Ambulation | 113 (77.4) | 26 (17.8) | 5 (3.4) | 2 (1.4) | 0 (0.0) | 0 (0.0) |
| Dexterity | 120 (82.2) | 16 (11.0) | 5 (3.4) | 4 (2.7) | 0 (0.0) | 1 (0.7) |
| Emotion | 37 (25.3) | 40 (27.4) | 52 (35.6) | 13 (8.9) | 4 (2.7) | - |
| Cognition | 68 (46.6) | 29 (19.9) | 28 (19.2) | 16 (11.0) | 5 (3.4) | 0 (0.0) |
| Pain | 45 (30.8) | 35 (24.0) | 37 (25.3) | 21 (14.4) | 8 (5.5) | - |
| EQ-5D-3L: Week 24 follow-up (n=136) | | | | | | |
| Mobility | 99 (72.8) | 36 (26.5) | 1 (0.7) | - | - | - |
| Self-care | 114 (83.8) | 22 (16.2) | 0 (0.0) | - | - | - |
| Usual Activities | 96 (70.6) | 38 (27.9) | 2 (1.5) | - | - | - |
| Pain/Discomfort | 61 (44.9) | 59 (43.4) | 16 (11.8) | - | - | - |
| Anxiety/Depression | 54 (39.7) | 67 (49.3) | 15 (11.0) | - | - | - |
| EQ-5D-5L: Week 24 follow-up (n=136) | | | | | | |
| Mobility | 93 (68.4) | 17 (12.5) | 16 (11.8) | 8 (5.9) | 2 (1.5) | - |
| Self-care | 113 (83.1) | 13 (9.6) | 6 (4.4) | 4 (2.9) | 0 (0.0) | - |
| Usual Activities | 84 (61.8) | 29 (21.3) | 16 (11.8) | 7 (5.1) | 0 (0.0) | - |
| Pain/Discomfort | 55 (40.4) | 34 (25.0) | 26 (19.1) | 15 (11) | 6 (4.4) | - |
| Anxiety/Depression | 52 (38.2) | 39 (28.7) | 28 (20.6) | 10 (7.4) | 7 (5.1) | - |
| HUI3: Week 24 follow-up (n=136) | | | | | | |
| Vision | 85 (62.5) | 41 (30.1) | 4 (2.9) | 3 (2.2) | 3 (2.2) | 0 (0.0) |
| Hearing | 117 (86.0) | 14 (10.3) | 3 (2.2) | 2 (1.5) | 0 (0.0) | 0 (0.0) |
| Speech | 120 (88.2) | 15 (11.0) | 0 (0.0) | 1 (0.7) | 0 (0.0) | - |
| Ambulation | 104 (76.5) | 25 (18.4) | 4 (2.9) | 2 (1.5) | 1 (0.7) | 0 (0.0) |
| Dexterity | 111 (81.6) | 17 (12.5) | 3 (2.2) | 4 (2.9) | 1 (0.7) | 0 (0.0) |
| Emotion | 46 (33.8) | 44 (32.4) | 26 (19.1) | 15 (11.0) | 5 (3.7) | - |
| Cognition | 87 (64.0) | 21 (15.4) | 13 (9.6) | 8 (5.9) | 7 (5.1) | 0 (0.0) |
| Pain | 47 (34.6) | 34 (25.0) | 32 (23.5) | 14 (10.3) | 9 (6.6) | - |
| HUI3 indicates Health Utilities Index Mark 3. | | | | | | |

**Supplementary Material 4**

| **SM4 Table A**. EQ-5D-3L, EQ-5D-5L and HUI3 change scores for ‘improved’ and ‘not improved’ groups, by external criterion, for participants satisfying the strict data quality criteria. Results are presented for three time periods. Values are means (standard deviations) unless stated otherwise.^a^ | | | |
| --- | --- | --- | --- |
| Time period | EQ-5D-3L | EQ-5D-5L | HUI3 |
| External criterion |  |  |  |
| Baseline to Week 12 |  |  |  |
| Beck Anxiety Inventory |  |  |  |
| Improved (n=37) | 0.195 (0.19) | 0.151 (0.18) | 0.193 (0.23) |
| Not improved (n=43) | 0.051 (0.16) | 0.077 (0.17) | 0.064 (0.23) |
| Difference (95% CI) | 0.144 (0.07, 0.22) | 0.073 (-0.01, 0.15) | 0.129 (0.03, 0.23) |
| Beck Depression Inventory-II |  |  |  |
| Improved (n=43) | 0.178 (0.20) | 0.173 (0.18) | 0.219 (0.21) |
| Not improved (n=36) | 0.041 (0.14) | 0.038 (0.15) | 0.007 (0.21) |
| Difference (95% CI) | 0.138 (0.06, 0.22) | 0.135 (0.06, 0.21) | 0.212 (0.127, 0.31) |
| Brief Pain Inventory ‒ pain intensity |  |  |  |
| Improved (n=27) | 0.201 (0.21) | 0.209 (0.19) | 0.231 (0.24) |
| Not improved (n=53) | 0.075 (0.16) | 0.062 (0.15) | 0.069 (0.21) |
| Difference (95% CI) | 0.126 (0.04, 0.21) | 0.147 (0.07, 0.22) | 0.163 (0.06, 0.27) |
| Brief Pain Inventory ‒ pain interference |  |  |  |
| Improved (n=24) | 0.192 (0.20) | 0.215 (0.17) | 0.235 (0.26) |
| Not improved (n=56) | 0.085 (0.17) | 0.067 (0.16) | 0.076 (0.21) |
| Difference (95% CI) | 0.106 (0.02, 0.19) | 0.148 (0.07, 0.23) | 0.159 (0.05, 0.27) |
| Brief Substance Craving Scale^b^ |  |  |  |
| Improved (n=55) | 0.126 (0.14) | 0.119 (0.15) | 0.139 (0.22) |
| Not improved (n=20) | 0.056 (0.24) | 0.066 (0.23) | 0.046 (0.23) |
| Difference (95% CI) | 0.070 (-0.02, 0.16) | 0.053 (-0.04, 0.15) | 0.093 (-0.02, 0.21) |
| Clinical Opiate Withdrawal Scale^c^ |  |  |  |
| Improved (n=35) | 0.142 (0.20) | 0.176 (0.19) | 0.190 (0.21) |
| Not improved (n=18) | 0.044 (0.17) | 0.045 (0.20) | 0.044 (0.21) |
| Difference (95% CI) | 0.098 (-0.01, 0.21) | 0.131 (0.02, 0.24) | 0.146 (0.02, 0.27) |
| Kessler Psychological Distress Scale |  |  |  |
| Improved (n=40) | 0.179 (0.18) | 0.156 (0.17) | 0.227 (0.21) |
| Not improved (n=40) | 0.056 (0.17) | 0.066 (0.18) | 0.021 (0.22) |
| Difference (95% CI) | 0.123 (0.04, 0.20) | 0.090 (0.01, 0.17) | 0.206 (0.11, 0.30) |
| EQ VAS (EQ-5D-5L) |  |  |  |
| Improved (n=35) | 0.175 (0.19) | 0.148 (0.18) | 0.217 (0.23) |
| Not improved (n=45) | 0.072 (0.17) | 0.082 (0.17) | 0.051 (0.22) |
| Difference (95% CI) | 0.103 (0.02, 0.18) | 0.066 (-0.01, 0.15) | 0.166 (0.07, 0.27) |
| Week 12 to Week 24 |  |  |  |
| Beck Anxiety Inventory |  |  |  |
| Improved (n=16) | 0.184 (0.21) | 0.133 (0.18) | 0.224 (0.22) |
| Not improved (n=53) | 0.018 (0.11) | 0.002 (0.12) | 0.016 (0.17) |
| Difference (95% CI) | 0.166 (0.09, 0.25) | 0.131 (0.06, 0.21) | 0.208 (0.11, 0.31) |
| Beck Depression Inventory-II |  |  |  |
| Improved (n=23) | 0.153 (0.20) | 0.088 (0.17) | 0.171 (0.23) |
| Not improved (n=46) | 0.008 (0.10) | 0.004 (0.12) | 0.011 (0.16) |
| Difference (95% CI) | 0.145 (0.07, 0.22) | 0.084 (0.01, 0.15) | 0.160 (0.06, 0.25) |
| Brief Pain Inventory ‒ pain intensity |  |  |  |
| Improved (n=14) | 0.151 (0.22) | 0.125 (0.18) | 0.133 (0.27) |
| Not improved (n=55) | 0.032 (0.13) | 0.009 (0.12) | 0.047 (0.18) |
| Difference (95% CI) | 0.119 (0.03, 0.21) | 0.116 (0.03, 0.20) | 0.086 (-0.03, 0.20) |
| Brief Pain Inventory ‒ pain interference |  |  |  |
| Improved (n=10) | 0.187 (0.25) | 0.157 (0.21) | 0.184 (0.25) |
| Not improved (n=59) | 0.034 (0.12) | 0.011 (0.12) | 0.044 (0.19) |
| Difference (95% CI) | 0.153 (0.05, 0.25) | 0.145 (0.05, 0.24) | 0.140 (0.01, 0.27) |
| Brief Substance Craving Scale^b^ |  |  |  |
| Improved (n=18) | 0.075 (0.21) | 0.095 (0.18) | 0.075 (0.21) |
| Not improved (n=41) | 0.046 (0.13) | 0.002 (0.12) | 0.071 (0.20) |
| Difference (95% CI) | 0.029 (-0.06, 0.12) | 0.093 (0.01, 0.17) | 0.004 (-0.11, 0.12) |
| Kessler Psychological Distress Scale |  |  |  |
| Improved (n=18) | 0.159 (0.20) | 0.122 (0.17) | 0.171 (0.23) |
| Not improved (n=51) | 0.020 (0.12) | 0.001 (0.12) | 0.026 (0.17) |
| Difference (95% CI) | 0.139 (0.06, 0.22) | 0.121 (0.05, 0.19) | 0.145 (0.04, 0.25) |
| EQ VAS (EQ-5D-5L) |  |  |  |
| Improved (n=48) | 0.148 (0.20) | 0.117 (0.19) | 0.159 (0.24) |
| Not improved (n=21) | 0.016 (0.12) | -0.005 (0.10) | 0.023 (0.17) |
| Difference (95% CI) | 0.132 (0.06, 0.21) | 0.122 (0.05, 0.19) | 0.136 (0.04, 0.24) |
| Baseline to Week 24 |  |  |  |
| Beck Anxiety Inventory |  |  |  |
| Improved (n=39) | 0.220 (0.24) | 0.196 (0.25) | 0.259 (0.25) |
| Not improved (n=38) | 0.057 (0.23) | 0.044 (0.21) | 0.041 (0.26) |
| Difference (95% CI) | 0.162 (0.06, 0.27) | 0.152 (0.05, 0.26) | 0.218 (0.10, 0.33) |
| Beck Depression Inventory-II |  |  |  |
| Improved (n=46) | 0.212 (0.23) | 0.188 (0.22) | 0.233 (0.27) |
| Not improved (n=31) | 0.032 (0.23) | 0.022 (0.24) | 0.029 (0.25) |
| Difference (95% CI) | 0.180 (0.07, 0.29) | 0.166 (0.06, 0.27) | 0.205 (0.08, 0.32) |
| Brief Pain Inventory ‒ pain intensity |  |  |  |
| Improved (n=25) | 0.245 (0.23) | 0.252 (0.23) | 0.308 (0.23) |
| Not improved (n=52) | 0.089 (0.24) | 0.058 (0.22) | 0.076 (0.27) |
| Difference (95% CI) | 0.156 (0.04, 0.27) | 0.194 (0.09, 0.30) | 0.232 (0.11, 0.36) |
| Brief Pain Inventory ‒ pain interference |  |  |  |
| Improved (n=27) | 0.269 (0.23) | 0.257 (0.25) | 0.314 (0.24) |
| Not improved (n=50) | 0.070 (0.23) | 0.047 (0.20) | 0.063 (0.26) |
| Difference (95% CI) | 0.199 (0.09, 0.31) | 0.210 (0.11, 0.31) | 0.250 (0.13, 0.37) |
| Brief Substance Craving Scale^b^ |  |  |  |
| Improved (n=53) | 0.132 (0.26) | 0.115 (0.25) | 0.176 (0.29) |
| Not improved (n=15) | 0.163 (0.23) | 0.116 (0.23) | 0.050 (0.27) |
| Difference (95% CI) | -0.032 (-0.18, 0.12) | -0.001 (-0.14, 0.14) | 0.126 (-0.04, 0.29) |
| Kessler Psychological Distress Scale |  |  |  |
| Improved (n=43) | 0.216 (0.22) | 0.187 (0.22) | 0.233 (0.27) |
| Not improved (n=33) | 0.044 (0.25) | 0.037 (0.25) | 0.049 (0.25) |
| Difference (95% CI) | 0.171 (0.06, 0.28) | 0.149 (0.04, 0.26) | 0.183 (0.06, 0.30) |
| EQ VAS (EQ-5D-5L) |  |  |  |
| Improved (n=43) | 0.222 (0.20) | 0.189 (0.20) | 0.254 (0.24) |
| Not improved (n=34) | 0.035 (0.27) | 0.035 (0.26) | 0.021 (0.27) |
| Difference (95% CI) | 0.188 (0.08, 0.29) | 0.154 (0.05, 0.26) | 0.233 (0.12, 0.35) |
| CI indicates confidence interval; HUI3, Health Utilities Index Mark 3; VAS, visual analogue scale. | | | |
| ^a^ Change scores calculated as the participant’s value at the latter time point minus the value at the former time point. The *difference* in change scores is the mean value in the ‘improved’ group minus the mean value in the ‘not improved’ group. Distribution-based methods were used to define the ‘improved’ and ‘not improved’ groups (described in the Methods section of the paper). | | | |
| ^b^ Follow-up for the Brief Substance Craving Scale was at week 10 and week 22 (paired with week 12 and week 24 index scores, respectively). | | | |
| ^c^ For the Clinical Opiate Withdrawal Scale criterion, changes in index scores from baseline to week 4 were compared with clinical change measured from treatment initiation (any time from baseline to week 2) to week 4. | | | |
| **SM4 Table B**. EQ-5D-3L, EQ-5D-5L and HUI3 change scores for ‘improved’ and ‘not improved’ groups, by external criterion, for participants satisfying the soft data quality criteria. Results are presented for three time periods. Values are means (standard deviations) unless stated otherwise.^a^ | | | |
| Time period | EQ-5D-3L | EQ-5D-5L | HUI3 |
| External criterion |  |  |  |
| Baseline to Week 12 |  |  |  |
| Beck Anxiety Inventory |  |  |  |
| Improved (n=58) | 0.201 (0.25) | 0.165 (0.22) | 0.206 (0.29) |
| Not improved (n=67) | 0.036 (0.20) | 0.075 (0.22) | 0.055 (0.25) |
| Difference (95% CI) | 0.164 (0.09, 0.24) | 0.090 (0.01, 0.17) | 0.151 (0.06, 0.25) |
| Beck Depression Inventory-II |  |  |  |
| Improved (n=63) | 0.187 (0.23) | 0.176 (0.21) | 0.230 (0.29) |
| Not improved (n=61) | 0.034 (0.21) | 0.056 (0.22) | 0.015 (0.22) |
| Difference (95% CI) | 0.154 (0.07, 0.23) | 0.120 (0.04, 0.20) | 0.216 (0.12, 0.31) |
| Brief Pain Inventory ‒ pain intensity |  |  |  |
| Improved (n=38) | 0.234 (0.24) | 0.237 (0.22) | 0.242 (0.27) |
| Not improved (n=87) | 0.060 (0.22) | 0.065 (0.21) | 0.074 (0.27) |
| Difference (95% CI) | 0.174 (0.09, 0.26) | 0.172 (0.09, 0.25) | 0.168 (0.06, 0.27) |
| Brief Pain Inventory ‒ pain interference |  |  |  |
| Improved (n=40) | 0.211 (0.24) | 0.237 (0.21) | 0.238 (0.30) |
| Not improved (n=85) | 0.066 (0.22) | 0.061 (0.205) | 0.072 (0.25) |
| Difference (95% CI) | 0.145 (0.06, 0.23) | 0.176 (0.10, 0.25) | 0.166 (0.06, 0.27) |
| Brief Substance Craving Scale^b^ |  |  |  |
| Improved (n=89) | 0.134 (0.23) | 0.131 (0.22) | 0.160 (0.27) |
| Not improved (n=27) | 0.020 (0.23) | 0.036 (0.21) | -0.002 (0.26) |
| Difference (95% CI) | 0.114 (0.01, 0.21) | 0.095 (0.00, 0.19) | 0.162 (0.04, 0.28) |
| Clinical Opiate Withdrawal Scale^c^ |  |  |  |
| Improved (n=55) | 0.115 (0.25) | 0.131 (0.22) | 0.170 (0.25) |
| Not improved (n=29) | 0.037 (0.19) | 0.011 (0.21) | 0.062 (0.24) |
| Difference (95% CI) | 0.077 (-0.03, 0.18) | 0.120 (0.02, 0.22) | 0.107 (-0.01, 0.22) |
| Kessler Psychological Distress Scale |  |  |  |
| Improved (n=55) | 0.202 (0.23) | 0.188 (0.22) | 0.270 (0.28) |
| Not improved (n=70) | 0.042 (0.22) | 0.061 (0.21) | 0.011 (0.21) |
| Difference (95% CI) | 0.160 (0.08, 0.24) | 0.127 (0.05, 0.20) | 0.259 (0.17, 0.35) |
| EQ VAS (EQ-5D-5L) |  |  |  |
| Improved (n=52) | 0.190 (0.24) | 0.183 (0.23) | 0.229 (0.29) |
| Not improved (n=73) | 0.057 (0.21) | 0.070 (0.21) | 0.051 (0.25) |
| Difference (95% CI) | 0.133 (0.05, 0.21) | 0.114 (0.04, 0.19) | 0.178 (0.08, 0.27) |
| Week 12 to Week 24 |  |  |  |
| Beck Anxiety Inventory |  |  |  |
| Improved (n=25) | 0.146 (0.21) | 0.111 (0.15) | 0.181 (0.24) |
| Not improved (n=78) | -0.005 (0.16) | -0.008 (0.15) | 0.002 (0.16) |
| Difference (95% CI) | 0.151 (0.07, 0.23) | 0.119 (0.05, 0.19) | 0.179 (0.09, 0.26) |
| Beck Depression Inventory-II |  |  |  |
| Improved (n=35) | 0.145 (0.18) | 0.077 (0.15) | 0.155 (0.23) |
| Not improved (n=68) | -0.027 (0.15) | -0.007 (0.15) | -0.011 (0.16) |
| Difference (95% CI) | 0.173 (0.11, 0.24) | 0.084 (0.02, 0.15) | 0.166 (0.09, 0.24) |
| Brief Pain Inventory ‒ pain intensity |  |  |  |
| Improved (n=23) | 0.062 (0.26) | 0.051 (0.23) | 0.094 (0.24) |
| Not improved (n=80) | 0.023 (0.16) | 0.013 (0.13) | 0.031 (0.19) |
| Difference (95% CI) | 0.039 (-0.05, 0.12) | 0.038 (-0.03, 0.11) | 0.063 (-0.03, 0.16) |
| Brief Pain Inventory ‒ pain interference |  |  |  |
| Improved (n=15) | 0.134 (0.23) | 0.135 (0.18) | 0.163 (0.21) |
| Not improved (n=88) | 0.014 (0.17) | 0.002 (0.14) | 0.025 (0.19) |
| Difference (95% CI) | 0.120 (0.02, 0.22) | 0.133 (0.05, 0.22) | 0.137 (0.03, 0.25) |
| Brief Substance Craving Scale^b^ |  |  |  |
| Improved (n=27) | 0.044 (0.21) | 0.097 (0.18) | 0.059 (0.19) |
| Not improved (n=61) | 0.016 (0.17) | -0.015 (0.14) | 0.044 (0.22) |
| Difference (95% CI) | 0.028 (-0.06, 0.11) | 0.112 (0.04, 0.18) | 0.014 (-0.08, 0.11) |
| Kessler Psychological Distress Scale |  |  |  |
| Improved (n=28) | 0.124 (0.21) | 0.108 (0.16) | 0.146 (0.24) |
| Not improved (n=74) | -0.003 (0.16) | -0.010 (0.14) | 0.007 (0.17) |
| Difference (95% CI) | 0.127 (0.05, 0.20) | 0.118 (0.05, 0.18) | 0.139 (0.05, 0.22) |
| EQ VAS (EQ-5D-5L) |  |  |  |
| Improved (n=37) | 0.100 (0.20) | 0.099 (0.16) | 0.144 (0.21) |
| Not improved (n=66) | -0.007 (0.16) | -0.023 (0.14) | -0.010 (0.17) |
| Difference (95% CI) | 0.107 (0.04, 0.18) | 0.122 (0.06, 0.18) | 0.154 (0.08, 0.23) |
| Baseline to Week 24 |  |  |  |
| Beck Anxiety Inventory |  |  |  |
| Improved (n=55) | 0.230 (0.25) | 0.222 (0.24) | 0.282 (0.27) |
| Not improved (n=61) | 0.021 (0.23) | 0.049 (0.21) | 0.072 (0.27) |
| Difference (95% CI) | 0.210 (0.12, 0.30) | 0.172 (0.09, 0.25) | 0.210 (0.11, 0.31) |
| Beck Depression Inventory-II |  |  |  |
| Improved (n=67) | 0.204 (0.25) | 0.201 (0.23) | 0.258 (0.29) |
| Not improved (n=49) | 0.006 (0.23) | 0.035 (0.22) | 0.052 (0.25) |
| Difference (95% CI) | 0.198 (0.11, 0.29) | 0.166 (0.08, 0.25) | 0.206 (0.10, 0.31) |
| Brief Pain Inventory ‒ pain intensity |  |  |  |
| Improved (n=39) | 0.235 (0.27) | 0.257 (0.24) | 0.328 (0.28) |
| Not improved (n=76) | 0.063 (0.24) | 0.066 (0.21) | 0.092 (0.26) |
| Difference (95% CI) | 0.172 (0.07, 0.27) | 0.190 (0.10, 0.28) | 0.235 (0.13, 0.34) |
| Brief Pain Inventory ‒ pain interference |  |  |  |
| Improved (n=38) | 0.245 (0.24) | 0.269 (0.24) | 0.336 (0.27) |
| Not improved (n=78) | 0.059 (0.25) | 0.064 (0.20) | 0.091 (0.27) |
| Difference (95% CI) | 0.186 (0.09, 0.28) | 0.205 (0.12, 0.29) | 0.244 (0.14, 0.35) |
| Brief Substance Craving Scale^b^ |  |  |  |
| Improved (n=81) | 0.123 (0.28) | 0.133 (0.24) | 0.191 (0.30) |
| Not improved (n=20) | 0.100 (0.24) | 0.070 (0.22) | 0.069 (0.27) |
| Difference (95% CI) | 0.024 (-0.11, 0.16) | 0.062 (-0.06, 0.18) | 0.123 (-0.02, 0.27) |
| Kessler Psychological Distress Scale |  |  |  |
| Improved (n=63) | 0.216 (0.24) | 0.215 (0.22) | 0.270 (0.28) |
| Not improved (n=52) | 0.002 (0.24) | 0.018 (0.22) | 0.046 (0.24) |
| Difference (95% CI) | 0.214 (0.13, 0.30) | 0.197 (0.12, 0.28) | 0.224 (0.13, 0.32) |
| EQ VAS (EQ-5D-5L) |  |  |  |
| Improved (n=66) | 0.191 (0.24) | 0.198 (0.21) | 0.249 (0.27) |
| Not improved (n=51) | 0.031 (0.26) | 0.039 (0.24) | 0.071 (0.28) |
| Difference (95% CI) | 0.160 (0.07, 0.25) | 0.158 (0.07, 0.24) | 0.177 (0.08, 0.28) |
| CI indicates confidence interval; HUI3, Health Utilities Index Mark 3; VAS, visual analogue scale. | | | |
| ^a^ Change scores calculated as the participant’s value at the latter time point minus the value at the former time point. The *difference* in change scores is the mean value in the ‘improved’ group minus the mean value in the ‘not improved’ group. Distribution-based methods were used to define the ‘improved’ and ‘not improved’ groups (described in the Methods section of the paper). | | | |
| ^b^ Follow-up for the Brief Substance Craving Scale was at week 10 and week 22 (paired with week 12 and week 24 index scores, respectively). | | | |
| ^c^ For the Clinical Opiate Withdrawal Scale criterion, changes in index scores from baseline to week 4 were compared with clinical change measured from treatment initiation (any time from baseline to week 2) to week 4. | | | |

| **SM4 Table C**. EQ-5D-3L, EQ-5D-5L and HUI3 change scores for ‘improved’ and ‘not improved’ groups, by external criterion, for the study sample when applying no data quality conditions. Results are presented for three time periods. Values are means (standard deviations) unless stated otherwise.^a^ | | | |
| --- | --- | --- | --- |
| Time period | EQ-5D-3L | EQ-5D-5L | HUI3 |
| External criterion |  |  |  |
| Baseline to Week 12 |  |  |  |
| Beck Anxiety Inventory |  |  |  |
| Improved (n=68) | 0.184 (0.24) | 0.159 (0.22) | 0.205 (0.27) |
| Not improved (n=78) | 0.013 (0.21) | 0.055 (0.23) | 0.025 (0.25) |
| Difference (95% CI) | 0.171 (0.10, 0.25) | 0.105 (0.03, 0.18) | 0.180 (0.09, 0.27) |
| Beck Depression Inventory-II |  |  |  |
| Improved (n=68) | 0.187 (0.23) | 0.168 (0.21) | 0.216 (0.29) |
| Not improved (n=77) | 0.007 (0.22) | 0.047 (0.23) | 0.013 (0.23) |
| Difference (95% CI) | 0.181 (0.11, 0.25) | 0.121 (0.05, 0.19) | 0.203 (0.12, 0.29) |
| Brief Pain Inventory ‒ pain intensity |  |  |  |
| Improved (n=45) | 0.203 (0.24) | 0.225 (0.22) | 0.244 (0.25) |
| Not improved (n=101) | 0.043 (0.23) | 0.049 (0.21) | 0.049 (0.27) |
| Difference (95% CI) | 0.160 (0.08, 0.24) | 0.176 (0.10, 0.25) | 0.195 (0.10, 0.29) |
| Brief Pain Inventory ‒ pain interference |  |  |  |
| Improved (n=46) | 0.178 (0.25) | 0.220 (0.22) | 0.229 (0.29) |
| Not improved (n=100) | 0.053 (0.23) | 0.050 (0.21) | 0.054 (0.25) |
| Difference (95% CI) | 0.125 (0.04, 0.21) | 0.170 (0.09, 0.25) | 0.176 (0.08, 0.27) |
| Brief Substance Craving Scale^b^ |  |  |  |
| Improved (n=101) | 0.113 (0.24) | 0.130 (0.21) | 0.150 (0.27) |
| Not improved (n=35) | 0.013 (0.22) | -0.001 (0.24) | -0.024 (0.26) |
| Difference (95% CI) | 0.10 (0.01, 0.19) | 0.131 (0.04, 0.22) | 0.175 (0.07, 0.28) |
| Clinical Opiate Withdrawal Scale^c^ |  |  |  |
| Improved (n=62) | 0.097 (0.25) | 0.130 (0.21) | 0.140 (0.27) |
| Not improved (n=35) | 0.045 (0.18) | 0.046 (0.22) | 0.088 (0.23) |
| Difference (95% CI) | 0.052 (-0.04, 0.15) | 0.083 (-0.01, 0.17) | 0.052 (-0.05, 0.16) |
| Kessler Psychological Distress Scale |  |  |  |
| Improved (n=63) | 0.193 (0.22) | 0.170 (0.22) | 0.244 (0.28) |
| Not improved (n=83) | 0.016 (0.23) | 0.053 (0.23) | 0.006 (0.22) |
| Difference (95% CI) | 0.178 (0.10, 0.25) | 0.116 (0.04, 0.19) | 0.238 (0.16, 0.32) |
| EQ VAS (EQ-5D-5L) |  |  |  |
| Improved (n=62) | 0.173 (0.24) | 0.166 (0.24) | 0.212 (0.28) |
| Not improved (n=84) | 0.033 (0.23) | 0.058 (0.21) | 0.033 (0.25) |
| Difference (95% CI) | 0.141 (0.06, 0.22) | 0.108 (0.03, 0.18) | 0.179 (0.09, 0.27) |
| Week 12 to Week 24 |  |  |  |
| Beck Anxiety Inventory |  |  |  |
| Improved (n=34) | 0.106 (0.22) | 0.082 (0.21) | 0.156 (0.27) |
| Not improved (n=88) | -0.008 (0.17) | -0.006 (0.15) | 0.003 (0.18) |
| Difference (95% CI) | 0.114 (0.04, 0.19) | 0.088 (0.02, 0.15) | 0.153 (0.07, 0.24) |
| Beck Depression Inventory-II |  |  |  |
| Improved (n=44) | 0.132 (0.19) | 0.061 (0.18) | 0.161 (0.23) |
| Not improved (n=77) | -0.035 (0.16) | -0.005 (0.17) | -0.016 (0.18) |
| Difference (95% CI) | 0.167 (0.10, 0.23) | 0.065 (0.00, 0.13) | 0.177 (0.10, 0.25) |
| Brief Pain Inventory ‒ pain intensity |  |  |  |
| Improved (n=25) | 0.065 (0.25) | 0.050 (0.22) | 0.096 (0.23) |
| Not improved (n=97) | 0.013 (0.17) | 0.010 (0.16) | 0.033 (0.21) |
| Difference (95% CI) | 0.053 (-0.03, 0.14) | 0.040 (-0.04, 0.12) | 0.063 (-0.03, 0.16) |
| Brief Pain Inventory ‒ pain interference |  |  |  |
| Improved (n=18) | 0.138 (0.21) | 0.148 (0.19) | 0.186 (0.23) |
| Not improved (n=104) | 0.004 (0.18) | -0.004 (0.16) | 0.021 (0.21) |
| Difference (95% CI) | 0.134 (0.04, 0.23) | 0.152 (0.07, 0.23) | 0.165 (0.06, 0.27) |
| Brief Substance Craving Scale^b^ |  |  |  |
| Improved (n=34) | 0.041 (0.22) | 0.083 (0.18) | 0.057 (0.2) |
| Not improved (n=70) | 0.009 (0.18) | -0.024 (0.16) | 0.045 (0.22) |
| Difference (95% CI) | 0.032 (-0.05, 0.11) | 0.107 (0.04, 0.18) | 0.012 (-0.08, 0.10) |
| Kessler Psychological Distress Scale |  |  |  |
| Improved (n=35) | 0.127 (0.19) | 0.086 (0.21) | 0.161 (0.24) |
| Not improved (n=86) | -0.019 (0.18) | -0.008 (0.15) | -0.002 (0.19) |
| Difference (95% CI) | 0.146 (0.07, 0.22) | 0.094 (0.03, 0.16) | 0.163 (0.08, 0.24) |
| EQ VAS (EQ-5D-5L) |  |  |  |
| Improved (n=45) | 0.075 (0.21) | 0.082 (0.20) | 0.133 (0.23) |
| Not improved (n=77) | -0.007 (0.18) | -0.019 (0.14) | -0.005 (0.19) |
| Difference (95% CI) | 0.082 (0.01, 0.15) | 0.101 (0.04, 0.16) | 0.138 (0.06, 0.21) |
| Baseline to Week 24 |  |  |  |
| Beck Anxiety Inventory |  |  |  |
| Improved (n=67) | 0.207 (0.24) | 0.209 (0.24) | 0.259 (0.26) |
| Not improved (n=68) | 0.009 (0.23) | 0.044 (0.23) | 0.061 (0.26) |
| Difference (95% CI) | 0.197 (0.12, 0.28) | 0.166 (0.09, 0.24) | 0.198 (0.11, 0.29) |
| Beck Depression Inventory-II |  |  |  |
| Improved (n=77) | 0.192 (0.24) | 0.209 (0.22) | 0.251 (0.27) |
| Not improved (n=58) | -0.006 (0.23) | 0.016 (0.23) | 0.038 (0.24) |
| Difference (95% CI) | 0.198 (0.12, 0.28) | 0.192 (0.11, 0.27) | 0.212 (0.12, 0.30) |
| Brief Pain Inventory ‒ pain intensity |  |  |  |
| Improved (n=43) | 0.223 (0.26) | 0.271 (0.24) | 0.320 (0.27) |
| Not improved (n=91) | 0.054 (0.23) | 0.057 (0.22) | 0.085 (0.25) |
| Difference (95% CI) | 0.170 (0.08, 0.26) | 0.214 (0.13, 0.30) | 0.236 (0.14, 0.33) |
| Brief Pain Inventory ‒ pain interference |  |  |  |
| Improved (n=42) | 0.232 (0.24) | 0.283 (0.24) | 0.327 (0.26) |
| Not improved (n=93) | 0.051 (0.24) | 0.055 (0.21) | 0.084 (0.26) |
| Difference (95% CI) | 0.182 (0.09, 0.27) | 0.228 (0.15, 0.31) | 0.243 (0.15, 0.34) |
| Brief Substance Craving Scale^b^ |  |  |  |
| Improved (n=93) | 0.110 (0.27) | 0.132 (0.24) | 0.181 (0.29) |
| Not improved (n=27) | 0.085 (0.24) | 0.064 (0.25) | 0.062 (0.25) |
| Difference (95% CI) | 0.025 (-0.09, 0.14) | 0.068 (-0.04, 0.17) | 0.119 (-0.00, 0.24) |
| Kessler Psychological Distress Scale |  |  |  |
| Improved (n=72) | 0.195 (0.24) | 0.207 (0.23) | 0.251 (0.28) |
| Not improved (n=62) | 0.004 (0.24) | 0.022 (0.23) | 0.048 (0.23) |
| Difference (95% CI) | 0.192 (0.11, 0.27) | 0.184 (0.11, 0.26) | 0.203 (0.11, 0.29) |
| EQ VAS (EQ-5D-5L) |  |  |  |
| Improved (n=76) | 0.168 (0.24) | 0.199 (0.22) | 0.236 (0.26) |
| Not improved (n=60) | 0.032 (0.25) | 0.028 (0.25) | 0.063 (0.28) |
| Difference (95% CI) | 0.135 (0.05, 0.22) | 0.172 (0.09, 0.25) | 0.172 (0.08, 0.26) |
| CI indicates confidence interval; HUI3, Health Utilities Index Mark 3; VAS, visual analogue scale. | | | |
| ^a^ Change scores calculated as the participant’s value at the latter time point minus the value at the former time point. The *difference* in change scores is the mean value in the ‘improved’ group minus the mean value in the ‘not improved’ group. Distribution-based methods were used to define the ‘improved’ and ‘not improved’ groups (described in the Methods section of the paper). | | | |
| ^b^ Follow-up for the Brief Substance Craving Scale was at week 10 and week 22 (paired with week 12 and week 24 index scores, respectively). | | | |
| ^c^ For the Clinical Opiate Withdrawal Scale criterion, changes in index scores from baseline to week 4 were compared with clinical change measured from treatment initiation (any time from baseline to week 2) to week 4. | | | |

**Supplementary Material 5**

| **SM5 Table A**. Descriptive statistics for the EQ-5D-3L, EQ-5D-5L and HUI3, by time point and time period, for participants satisfying the soft data quality criteria. | | | | | | | | | |
| --- | --- | --- | --- | --- | --- | --- | --- | --- | --- |
| Instrument | n | Mean | SD | Median | IQR | Full health  (%) | Negative score (%) | Max. | Min. |
| Time point/period |  |  |  |  |  |  |  |  |  |
| EQ-5D-3L |  |  |  |  |  |  |  |  |  |
| Baseline | 149 | 0.630^a^ | 0.245 | 0.709 | 0.289 | 8.1 | 1.3 | 1.000 | -0.187 |
| Week 12 follow-up | 133 | 0.740^a^ | 0.211 | 0.781 | 0.189 | 20.3 | 0.0 | 1.000 | 0.122 |
| Week 24 follow-up | 125 | 0.775^a^ | 0.212 | 0.781 | 0.299 | 28.0 | 0.8 | 1.000 | -0.154 |
| Change score (12W-B) | 125 | 0.113 | 0.236 | 0.072 | 0.253 | - | - | 1.013 | -0.541 |
| Change score (24W-12W) | 103 | 0.032 | 0.182 | 0.000 | 0.118 | - | - | 0.807 | -0.689 |
| Change score (24W-B) | 117 | 0.121^a^ | 0.259 | 0.091 | 0.288 | - | - | 0.935 | -0.600 |
| EQ-5D-5L |  |  |  |  |  |  |  |  |  |
| Baseline | 149 | 0.637^a^ | 0.251 | 0.707 | 0.352 | 5.4 | 0.7 | 0.949 | -0.066 |
| Week 12 follow-up | 133 | 0.754^a^ | 0.209 | 0.829 | 0.204 | 9.8 | 0.0 | 0.949 | 0.038 |
| Week 24 follow-up | 125 | 0.787^a^ | 0.217 | 0.867 | 0.185 | 26.4 | 0.8 | 0.949 | -0.044 |
| Change score (12W-B) | 125 | 0.117 | 0.222 | 0.065 | 0.235 | - | - | 0.845 | -0.603 |
| Change score (24W-12W) | 103 | 0.021 | 0.155 | 0.005 | 0.128 | - | - | 0.689 | -0.561 |
| Change score (24W-B) | 117 | 0.129^a^ | 0.239 | 0.101 | 0.248 | - | - | 0.777 | -0.667 |
| HUI3 |  |  |  |  |  |  |  |  |  |
| Baseline | 149 | 0.491^b,c^ | 0.315 | 0.526 | 0.445 | 4.0 | 9.4 | 1.000 | -0.257 |
| Week 12 follow-up | 133 | 0.618^b,c^ | 0.312 | 0.680 | 0.467 | 7.5 | 5.3 | 1.000 | -0.243 |
| Week 24 follow-up | 125 | 0.697^b,c^ | 0.310 | 0.756 | 0.423 | 20.8 | 4.0 | 1.000 | -0.298 |
| Change score (12W-B) | 125 | 0.125 | 0.278 | 0.104 | 0.296 | - | - | 0.997 | -0.499 |
| Change score (24W-12W) | 103 | 0.045 | 0.2 | 0.027 | 0.191 | - | - | 0.707 | -0.399 |
| Change score (24W-B) | 117 | 0.171^b,c^ | 0.289 | 0.137 | 0.363 | - | - | 0.897 | -0.505 |
| 12W indicates 12-week follow-up; 24W, 24-week follow-up; B, baseline; HUI3, Health Utilities Index Mark 3; IQR, interquartile range; max., maximum; min., minimum; SD, standard deviation. | | | | | | | | | |
| ^a^ Statistically different to the corresponding HUI3 index/change score using a significance level of 0.05 (paired t-test). | | | | | | | | | |
| ^b^ Statistically different to the corresponding EQ-5D-3L index/change score using a significance level of 0.05 (paired t-test). | | | | | | | | | |
| ^c^ Statistically different to the corresponding EQ-5D-5L index/change score using a significance level of 0.05 (paired t-test). | | | | | | | | | |

| **SM5 Table B**. Descriptive statistics for the EQ-5D-3L, EQ-5D-5L and HUI3, by time point and time period, for the study sample when applying no data quality conditions. | | | | | | | | | |
| --- | --- | --- | --- | --- | --- | --- | --- | --- | --- |
| Instrument | n | Mean | SD | Median | IQR | Full health  (%) | Negative score (%) | Max. | Min. |
| Time point/period |  |  |  |  |  |  |  |  |  |
| EQ-5D-3L |  |  |  |  |  |  |  |  |  |
| Baseline | 160 | 0.636^a^ | 0.242 | 0.709 | 0.289 | 8.1 | 1.3 | 1.000 | -0.187 |
| Week 12 follow-up | 146 | 0.726^a^ | 0.213 | 0.781 | 0.248 | 18.5 | 0.0 | 1.000 | 0.122 |
| Week 24 follow-up | 136 | 0.753^a^ | 0.232 | 0.781 | 0.337 | 25.7 | 0.7 | 1.000 | -0.154 |
| Change score (12W-B) | 146 | 0.092 | 0.24 | 0.063 | 0.265 | - | - | 1.013 | -0.541 |
| Change score (24W-12W) | 122 | 0.023 | 0.191 | 0.000 | 0.160 | - | - | 0.807 | -0.689 |
| Change score (24W-B) | 136 | 0.108^a^ | 0.253 | 0.072 | 0.256 | - | - | 0.935 | -0.600 |
| EQ-5D-5L |  |  |  |  |  |  |  |  |  |
| Baseline | 160 | 0.635^a^ | 0.250 | 0.707 | 0.373 | 5.0 | 0.6 | 0.949 | -0.066 |
| Week 12 follow-up | 146 | 0.741^a^ | 0.219 | 0.823 | 0.223 | 8.9 | 0.0 | 0.949 | 0.038 |
| Week 24 follow-up | 136 | 0.764^a^ | 0.234 | 0.847 | 0.214 | 25.0 | 0.7 | 0.949 | -0.044 |
| Change score (12W-B) | 146 | 0.104 | 0.228 | 0.061 | 0.255 | - | - | 0.845 | -0.603 |
| Change score (24W-12W) | 122 | 0.018 | 0.171 | 0.016 | 0.132 | - | - | 0.689 | -0.593 |
| Change score (24W-B) | 136 | 0.124 | 0.246 | 0.086 | 0.261 | - | - | 0.777 | -0.667 |
| HUI3 |  |  |  |  |  |  |  |  |  |
| Baseline | 160 | 0.497^b,c^ | 0.315 | 0.535 | 0.475 | 3.8 | 9.4 | 1.000 | -0.257 |
| Week 12 follow-up | 146 | 0.604^b,c^ | 0.310 | 0.658 | 0.472 | 6.8 | 4.8 | 1.000 | -0.243 |
| Week 24 follow-up | 136 | 0.667^b,c^ | 0.329 | 0.748 | 0.452 | 19.1 | 5.1 | 1.000 | -0.312 |
| Change score (12W-B) | 146 | 0.109 | 0.276 | 0.090 | 0.306 | - | - | 0.997 | -0.603 |
| Change score (24W-12W) | 122 | 0.046 | 0.217 | 0.027 | 0.206 | - | - | 0.707 | -0.399 |
| Change score (24W-B) | 136 | 0.160^b^ | 0.277 | 0.122 | 0.344 | - | - | 0.897 | -0.505 |
| 12W indicates 12-week follow-up; 24W, 24-week follow-up; B, baseline; HUI3, Health Utilities Index Mark 3; IQR, interquartile range; max., maximum; min., minimum; SD, standard deviation. | | | | | | | | | |
| ^a^ Statistically different to the corresponding HUI3 index/change score using a significance level of 0.05 (paired t-test). | | | | | | | | | |
| ^b^ Statistically different to the corresponding EQ-5D-3L index/change score using a significance level of 0.05 (paired t-test). | | | | | | | | | |
| ^c^ Statistically different to the corresponding EQ-5D-5L index/change score using a significance level of 0.05 (paired t-test). | | | | | | | | | |

**Supplementary Material 6**

| **SM6 Table A.** Area under the receiver operating characteristics (ROC) curve analysis, by preference-based instrument and time period, for participants satisfying the soft data quality criteria.^a^ | | | | |
| --- | --- | --- | --- | --- |
| Time period |  | Area under the ROC curve (95% confidence interval) | | |
| External criterion | n | EQ-5D-3L | EQ-5D-5L | HUI3 |
| Baseline to Week 12 |  |  |  |  |
| Beck Anxiety Inventory | 125 | 0.695 (0.60, 0.79) | 0.618 (0.52, 0.72)^c^ | 0.654 (0.56, 0.75) |
| Beck Depression Inventory-II | 124 | 0.697 (0.60, 0.79) | 0.676 (0.58, 0.77) | 0.720 (0.63, 0.81) |
| Brief Pain Inventory ‒ pain intensity | 125 | 0.697 (0.59, 0.80) | 0.731 (0.64, 0.83)^b^ | 0.683 (0.58, 0.78) |
| Brief Pain Inventory ‒ pain interference | 125 | 0.664 (0.56, 0.77) | 0.735 (0.64, 0.83)^b^ | 0.648 (0.54, 0.75) |
| Brief Substance Craving Scale^d^ | 116 | 0.643 (0.52, 0.77) | 0.649 (0.52, 0.78) | 0.650 (0.52, 0.78) |
| Clinical Opiate Withdrawal Scale^e^ | 84 | 0.574 (0.45, 0.70)^c^ | 0.669 (0.55, 0.79) | 0.648 (0.52, 0.78) |
| Kessler Psychological Distress Scale | 125 | 0.707 (0.62, 0.80) | 0.678 (0.58, 0.77) | 0.784 (0.70, 0.87)^b^ |
| EQ VAS (EQ-5D-5L) | 125 | 0.659 (0.56, 0.76) | 0.625 (0.52, 0.73) | 0.680 (0.58, 0.77) |
| Week 12 to Week 24 |  |  |  |  |
| Beck Anxiety Inventory | 103 | 0.723 (0.61, 0.84) | 0.723 (0.61, 0.83) | 0.726 (0.61, 0.85) |
| Beck Depression Inventory-II | 103 | 0.777 (0.68, 0.88)^b^ | 0.667 (0.56, 0.78) | 0.718 (0.61, 0.83) |
| Brief Pain Inventory ‒ pain intensity | 103 | 0.544 (0.41, 0.68)^c^ | 0.577 (0.43, 0.72)^c^ | 0.574 (0.43, 0.72)^c^ |
| Brief Pain Inventory ‒ pain interference | 103 | 0.656 (0.50, 0.81) | 0.738 (0.60, 0.87)^b^ | 0.684 (0.53, 0.84) |
| Brief Substance Craving Scale^d^ | 88 | 0.508 (0.38, 0.63)^c^ | 0.656 (0.53, 0.78) | 0.522 (0.39, 0.65)^c^ |
| Kessler Psychological Distress Scale | 102 | 0.708 (0.59, 0.82) | 0.714 (0.60, 0.82) | 0.661 (0.54, 0.78) |
| EQ VAS (EQ-5D-5L) | 103 | 0.663 (0.55, 0.77) | 0.729 (0.62, 0.83) | 0.717 (0.61, 0.82) |
| Baseline to Week 24 |  |  |  |  |
| Beck Anxiety Inventory | 116 | 0.747 (0.66, 0.84)^b^ | 0.703 (0.61, 0.80) | 0.715 (0.62, 0.81) |
| Beck Depression Inventory-II | 116 | 0.717 (0.62, 0.81) | 0.709 (0.61, 0.81) | 0.684 (0.59, 0.78) |
| Brief Pain Inventory ‒ pain intensity | 115 | 0.661 (0.55, 0.77) | 0.726 (0.63, 0.82) | 0.726 (0.63, 0.82) |
| Brief Pain Inventory ‒ pain interference | 116 | 0.702 (0.60, 0.81) | 0.765 (0.67, 0.86)^b^ | 0.754 (0.66, 0.85)^b^ |
| Brief Substance Craving Scale^d^ | 101 | 0.544 (0.40, 0.69)^c^ | 0.586 (0.44, 0.73)^c^ | 0.617 (0.47, 0.76)^c^ |
| Kessler Psychological Distress Scale | 115 | 0.751 (0.66, 0.84)^b^ | 0.761 (0.67, 0.85)^b^ | 0.703 (0.61, 0.80) |
| EQ VAS (EQ-5D-5L) | 117 | 0.682 (0.58, 0.78) | 0.688 (0.59, 0.79) | 0.684 (0.59, 0.78) |
| CI indicates confidence interval; HUI3, Health Utilities Index Mark 3; VAS, visual analogue scale. | | | | |
| ^a^ Sample size variation within a time period is because of missing data for the external criteria. | | | | |
| ^b^ One of the highest ten area under the ROC curve estimates (across all 66 analyses). | | | | |
| ^c^ One of the lowest ten area under the ROC curve estimates (across all 66 analyses). | | | | |
| ^d^ Follow-up for the Brief Substance Craving Scale was at week 10 and week 22 (paired with week 12 and week 24 index scores, respectively). | | | | |
| ^e^ For the Clinical Opiate Withdrawal Scale criterion, changes in index scores from baseline to week 4 were compared with clinical change measured from treatment initiation (any time from baseline to week 2) to week 4. | | | | |

| **SM6 Table B.** Area under the receiver operating characteristics (ROC) curve analysis, by preference-based instrument and time period, for the study sample when applying no data quality conditions.^a^ | | | | |
| --- | --- | --- | --- | --- |
| Time period |  | Area under the ROC curve (95% confidence interval) | | |
| External criterion | n | EQ-5D-3L | EQ-5D-5L | HUI3 |
| Baseline to Week 12 |  |  |  |  |
| Beck Anxiety Inventory | 146 | 0.700 (0.62, 0.78) | 0.635 (0.54, 0.73) | 0.696 (0.61, 0.78) |
| Beck Depression Inventory-II | 145 | 0.727 (0.65, 0.81) | 0.665 (0.58, 0.75) | 0.701 (0.62, 0.79) |
| Brief Pain Inventory ‒ pain intensity | 146 | 0.633 (0.53, 0.73) | 0.717 (0.63, 0.81) | 0.666 (0.57, 0.76) |
| Brief Pain Inventory ‒ pain interference | 146 | 0.678 (0.58, 0.77) | 0.732 (0.64, 0.82) | 0.726 (0.64, 0.81) |
| Brief Substance Craving Scale^d^ | 136 | 0.630 (0.52, 0.74) | 0.675 (0.56, 0.79) | 0.668 (0.56, 0.78) |
| Clinical Opiate Withdrawal Scale^e^ | 97 | 0.540 (0.42, 0.66)^c^ | 0.623 (0.50, 0.74) | 0.570 (0.45, 0.69)^c^ |
| Kessler Psychological Distress Scale | 146 | 0.725 (0.64, 0.81) | 0.653 (0.56, 0.74) | 0.755 (0.67, 0.84)^b^ |
| EQ VAS (EQ-5D-5L) | 146 | 0.665 (0.58, 0.75) | 0.623 (0.53, 0.72) | 0.684 (0.60, 0.77) |
| Week 12 to Week 24 |  |  |  |  |
| Beck Anxiety Inventory | 122 | 0.664 (0.55, 0.78) | 0.654 (0.54, 0.77) | 0.682 (0.57, 0.79) |
| Beck Depression Inventory-II | 121 | 0.766 (0.68, 0.86)^b^ | 0.647 (0.54, 0.75) | 0.735 (0.64, 0.83)^b^ |
| Brief Pain Inventory ‒ pain intensity | 122 | 0.572 (0.45, 0.69)^c^ | 0.575 (0.44, 0.71)^c^ | 0.587 (0.46, 0.72)^c^ |
| Brief Pain Inventory ‒ pain interference | 122 | 0.689 (0.56, 0.82) | 0.735 (0.61, 0.85)^b^ | 0.711 (0.58, 0.84) |
| Brief Substance Craving Scale^d^ | 104 | 0.544 (0.43, 0.66)^c^ | 0.640 (0.53, 0.76) | 0.529 (0.41, 0.65)^c^ |
| Kessler Psychological Distress Scale | 121 | 0.725 (0.63, 0.82) | 0.668 (0.56, 0.77) | 0.690 (0.59, 0.79) |
| EQ VAS (EQ-5D-5L) | 122 | 0.605 (0.50, 0.71)^c^ | 0.688 (0.59, 0.79) | 0.675 (0.58, 0.77) |
| Baseline to Week 24 |  |  |  |  |
| Beck Anxiety Inventory | 135 | 0.737 (0.65, 0.82)^b^ | 0.689 (0.60, 0.78) | 0.716 (0.63, 0.80) |
| Beck Depression Inventory-II | 135 | 0.727 (0.64, 0.81) | 0.735 (0.65, 0.82)^b^ | 0.707 (0.62, 0.79) |
| Brief Pain Inventory ‒ pain intensity | 134 | 0.665 (0.57, 0.76) | 0.752 (0.66, 0.84)^b^ | 0.739 (0.65, 0.83)^b^ |
| Brief Pain Inventory ‒ pain interference | 135 | 0.700 (0.60, 0.80) | 0.785 (0.70, 0.87)^b^ | 0.766 (0.68, 0.85)^b^ |
| Brief Substance Craving Scale^d^ | 120 | 0.556 (0.42, 0.69)^c^ | 0.578 (0.45, 0.71)^c^ | 0.626 (0.50, 0.75) |
| Kessler Psychological Distress Scale | 134 | 0.727 (0.64, 0.81) | 0.737 (0.65, 0.82)^b^ | 0.695 (0.61, 0.78) |
| EQ VAS (EQ-5D-5L) | 136 | 0.661 (0.57, 0.75) | 0.696 (0.61, 0.79) | 0.686 (0.60, 0.78) |
| CI indicates confidence interval; HUI3, Health Utilities Index Mark 3; VAS, visual analogue scale. | | | | |
| ^a^ Sample size variation within a time period is because of missing data for the external criteria. | | | | |
| ^b^ One of the highest ten area under the ROC curve estimates (across all 66 analyses). Three estimates were tied for ninth highest area under the ROC curve. | | | | |
| ^c^ One of the lowest ten area under the ROC curve estimates (across all 66 analyses). | | | | |
| ^d^ Follow-up for the Brief Substance Craving Scale was at week 10 and week 22 (paired with week 12 and week 24 index scores, respectively). | | | | |
| ^e^ For the Clinical Opiate Withdrawal Scale criterion, changes in index scores from baseline to week 4 were compared with clinical change measured from treatment initiation (any time from baseline to week 2) to week 4. | | | | |
